# An In Silico Analysis of Genetic Variants and Structural Modeling of the Human Frataxin Protein in Friedreich’s Ataxia

**DOI:** 10.3390/ijms25115796

**Published:** 2024-05-26

**Authors:** Loiane Mendonça Abrantes Da Conceição, Lucio Mendes Cabral, Gabriel Rodrigues Coutinho Pereira, Joelma Freire De Mesquita

**Affiliations:** 1 Laboratory of Bioinformatics and Computational Biology, Federal University of the State of Rio de Janeiro (UNIRIO), Avenida Pasteur, 296, Urca, Rio de Janeiro 22290-250, Braziljoelma.mesquita@unirio.br (J.F.D.M.); 2 Pharmaceutical Industrial Technology Laboratory, Federal University of Rio de Janeiro (UFRJ), Avenida Carlos Chagas Filho, 373, Cidade Universitária, Rio de Janeiro 21941-590, Brazil; 3 Laboratory of Molecular Modeling & QSAR, Federal University of Rio de Janeiro (UFRJ), Avenida Carlos Chagas Filho, 373, Cidade Universitária, Rio de Janeiro 21941-590, Brazil

**Keywords:** frataxin, Friedreich’s ataxia, in silico analysis

## Abstract

Friedreich’s Ataxia (FRDA) stands out as the most prevalent form of hereditary ataxias, marked by progressive movement ataxia, loss of vibratory sensitivity, and skeletal deformities, severely affecting daily functioning. To date, the only medication available for treating FRDA is Omaveloxolone (Skyclarys^®^), recently approved by the FDA. Missense mutations within the human frataxin (FXN) gene, responsible for intracellular iron homeostasis regulation, are linked to FRDA development. These mutations induce FXN dysfunction, fostering mitochondrial iron accumulation and heightened oxidative stress, ultimately triggering neuronal cell death pathways. This study amalgamated 226 FXN genetic variants from the literature and database searches, with only 18 previously characterized. Predictive analyses revealed a notable prevalence of detrimental and destabilizing predictions for FXN mutations, predominantly impacting conserved residues crucial for protein function. Additionally, an accurate, comprehensive three-dimensional model of human FXN was constructed, serving as the basis for generating genetic variants I154F and W155R. These variants, selected for their severe clinical implications, underwent molecular dynamics (MD) simulations, unveiling flexibility and essential dynamic alterations in their N-terminal segments, encompassing FXN42, FXN56, and FXN78 domains pivotal for protein maturation. Thus, our findings indicate potential interaction profile disturbances in the FXN42, FXN56, and FXN78 domains induced by I154F and W155R mutations, aligning with the existing literature.

## 1. Introduction

Friedreich’s Ataxia (FRDA) is an autosomal recessive and neurodegenerative genetic disease, representing the most prevalent form among hereditary ataxias, affecting approximately 1 in every 50,000 individuals worldwide [1]. FRDA manifests with progressive ataxia of body movements, mainly affecting the lower limbs, together with decreased vibratory sensation, muscle loss, and increased susceptibility to the development of diabetes and hypertrophic cardiomyopathy. Furthermore, vestibular and auditory changes may be present, along with skeletal deformities such as scoliosis and cavus foot [2,3]. The onset of symptoms generally occurs around twenty years of age, leading to patients’ gradual loss of independence and impairing their ability to carry out daily activities, including occupational tasks [4].

To date, there is no cure for Friedreich’s ataxia (FRDA); some treatments are still in the study phase, such as omaveloxolone, an Nrf2 activator, aiming to improve mitochondrial function and restore the redox balance caused in FRDA [5,6]. Approximately 80% of rare diseases have a genetic origin, highlighting the importance of integrating this topic into social relationships, health, and research efforts, thus contributing to the exploration of new therapeutic and pharmaceutical interventions [7].

The human frataxin protein (FXN) comprises 210 amino acids and is predominantly located in the inner membrane of mitochondria [1]. Its main role involves the regulation of intracellular iron homeostasis, thus facilitating the biosynthesis of the heme group and assisting in the assembly and repair of iron–sulfur cluster (ISC) formations [8]. Missense mutations in the FXN gene result in the loss of frataxin function [9], leading to the dysregulation of iron metabolism and subsequent accumulation and precipitation of iron in mitochondria. This accumulated iron reacts with hydrogen peroxide generated by the respiratory chain, triggering oxidation and the production of highly reactive hydroxyl radicals (Fenton reaction), which disturb the redox balance. Furthermore, deficiencies in proteins containing iron–sulfur clusters impact electron transport and oxidative phosphorylation in the respiratory chain, amplifying the generation of free radicals and reactive oxygen species. Ultimately, oxidative stress-induced mitochondrial damage and the activation of apoptotic pathways occur in affected neurons [10].

Despite the discovery of numerous mutations through next-generation sequencing methods, experimental characterization of variants remains expensive, time-consuming, and difficult to implement. Computer simulations, or in silico approaches, offer a more efficient, faster, and cost-effective means of studying and predicting mutation effects. These simulations help prioritize potentially deleterious mutations for future wet-bench investigations. Furthermore, in silico prediction helps in the study of mutations associated with diseases, guiding the development of more effective drugs for treatment and improving the understanding of the molecular mechanisms underlying associated pathologies [11,12,13,14].

Although the complete structure of human FXN remains experimentally undetermined, the critical functional regions remain unknown [15]. Knowledge of three-dimensional structures contributes significantly to the understanding of biological activity and protein–ligand interactions, including drug interactions. Three-dimensional protein structures serve as fundamental starting points for rational drug design, a fundamental approach to contemporary drug discovery and pharmaceutical advancements. This strategy allows for faster and more efficient lead identification and optimization compared to traditional trial-and-error methods [16]. However, experimental determination of protein structures (wet-lab methods) remains expensive, time-consuming, and technically challenging. In this context, in silico structural prediction methods have emerged as valuable tools for fast, efficient, and accurate modeling of protein structures [13,17,18].

Despite the numerous advantages of dry-lab approaches, it is important to acknowledge that, like any other method, computational approaches also have inherent limitations. In the field of computational biology, algorithms may be constrained by the assumptions and simplifications intrinsic to the models. Additionally, the accuracy of these algorithms relies heavily on the quality of the experimental data used during the training and validation steps [19,20,21]. In this context, the efficiency and valuable insights provided by bioinformatics tools are indispensable allies of traditional methods, yet they still require further support from experimental approaches [22].

To overcome these limitations, we implemented several strategies that were effective within the frameworks previously established by our research group [11,12,23]. These strategies include using robust algorithms, integrating diverse modeling approaches, and comparing the outcomes with existing experimental data [24,25]. Building on this foundation, an integrated suite of methods was employed in this study to thoroughly characterize the structural and functional effects of mutations in the human frataxin protein. This framework included functional and stability prediction, evolutionary conservation analysis, model construction, and validation.

## 2. Results

### 2.1. Protein Sequence and Variant Acquisition

The native FXN sequence obtained from UniProt is complete, comprising 210 amino acids [26]. FXN is initially encoded in the nucleus and subsequently targeted to the mitochondria. This process occurs in two steps and depends on the action of the mitochondrial processing enzyme peptidase (MPP). Initially, this enzyme cleaves the transit peptide (residues 1–41), responsible for mitochondrial targeting, located in the N-terminal portion of frataxin, removing it and generating an intermediate form of the enzyme (residues 42–210). Subsequently, the intermediate form of frataxin can be cleaved at three different sites, giving rise to distinct mature forms of the protein: FXN56, FXN78, and mFXN (FXN81-210) [27].

**Figure 1:**
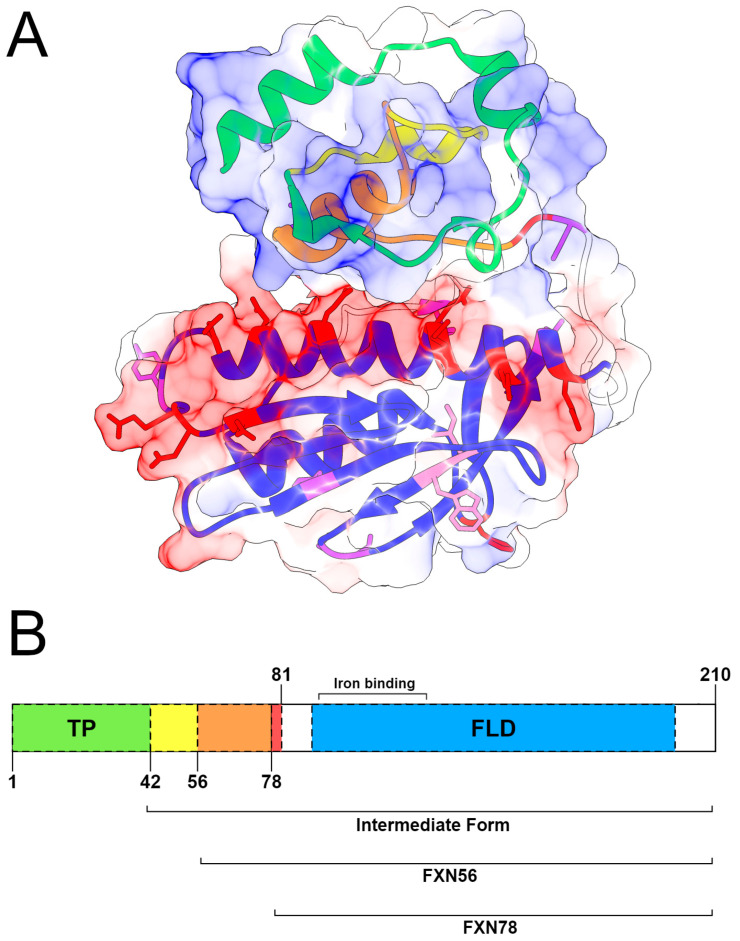
Three-dimensional structure and schematic representation of frataxin domains. (**A**) Three-dimensional structure of the complete human frataxin (FXN) protein showing the spatial arrangement of functional domains and key residues. Residues involved in iron binding and phosphorylation are depicted as sticks, colored red and purple, respectively. Notably, residues I154 and W155, involved in Friedreich ataxia mutations, are highlighted with pink sticks. Coulombic surface representation was displayed alongside the illustration to depict the charge composition of the surrounding areas, which illustrates the electrostatic potential. (**B**) Schematic representation of FXN designed using IBS software Version 1.0. (http://ibs.biocuckoo.org/, 05 April 2024). The native sequence of the protein is shown alongside functional domains and residues relevant to its function. The transit peptide (TP) is colored green, while the frataxin-like domain (FLD) is colored blue. The regions comprising the intermediate forms, FXN56 and FXN78, are enclosed in square brackets and highlighted in yellow, orange, and red, respectively. The mature form of the protein, mFXN, is indicated by the bracket from amino acids 81 to 210. The region densely populated with iron-binding residues is shown between brackets.

The mature forms of FXN are named after the position of the second cleavage by the MPP enzyme. A study by Schmucker et al., 2008, suggests that the predominant form of the enzyme is mFXN. The other mature forms, FXN56 (residues 56–210) and FXN78 (residues 78–210), are produced in an abnormal maturation process, and their physiological relevance is not precisely known [27]. The different regions involved in frataxin processing are illustrated in Figure 1.

According to Pfam, the frataxin protein possesses a conserved domain known as the frataxin-like domain (FLD), which spans residues 90 to 198. This domain is found in eukaryotic frataxins and the frataxin homologous protein, CyaY of *E. coli* [27]. Similar to frataxin, CyaY is a protein involved in the synthesis of iron–sulfur clusters and iron metabolism [26]. Given that iron accumulates in mitochondria in the absence or presence of defects in the frataxin protein, frataxin is believed to play a central role in iron homeostasis. Although iron was not found bound in protein structures experimentally determined for frataxin, it is believed that the binding of this protein to iron is essential for it to fulfill its biological function [28]. According to Sirano et al. (2000) and Huang et al. (2008), the following frataxin residues can bind to iron: E92, E96, E100, E101, E108, D112, D115, E121, D122, D124, and H177 [28,29,30].

The frataxin protein can be phosphorylated by tyrosine kinase, mainly at the Y118 site, leading to subsequent ubiquitination of the protein, a signal for its degradation. Furthermore, eight other phosphorylation sites were identified in the protein, as shown in Figure 1A, including S72, S81, T94, Y118, Y143, S160, S161, and Y205. Furthermore, the K171 position of frataxin is an acetylation site, while the K197 position is a sumoylation site [31,32,33].

Overall, 226 genetic variants of human FXN were compiled (Table S1), most of which have not yet been characterized in terms of their effects. Only 18 mutations were characterized in the literature and databases consulted. Ten mutations are deleterious: M1I, R40C, L106S, G130A, G130V, N146K, I154V, I154F, W155R, and W173G, while eight mutations are neutral: A14E, L33V, T44N, T44I, R60H, M76V, D178E, and D209G [34]. All the mutations identified as deleterious are associated with the development of Friedreich’s Ataxia (FRDA) [26]. In this regard, 208 mutations have not yet had their effects determined, comprising the vast majority of known human FXN mutations. The compiled mutations are evenly distributed throughout the protein sequence, similarly affecting the functional domains of FXN. This article presents the most comprehensive compilation of frataxin variants available in the literature to date.

The mutations occurring in relevant locations of the protein were verified, such as those occurring in iron-binding residues, where the following mutations were found: E92K, E96K, E100A, E108V, E108D, D112H, D112Y, D112A, D115E, D122Y, and H177Y. On the other hand, the mutations S81T, T94A, Y118C, S160C, S160T, and S160R are at important phosphorylation sites. Furthermore, the mutations K171E and K171R occur in the acetylation site and K197R in the sumoylation site of FXN.

### 2.2. Predictive Analysis

In this step, ten functional prediction algorithms were used to predict the neutral or deleterious effects of missense mutations on the FXN function (Table S2). Among them, PolyPhen-2, SIFT, PANTHER, and SNAP2 achieved 100% accuracy among the 10 known deleterious mutations, followed by SNAP with 90%, PredictSNP, SNP&GO, and PMut with 80%. On the other hand, the PhD-SNP and Mutpred2 algorithms achieved 70% and 30% accuracy in their predictions, respectively.

**Figure 2:**
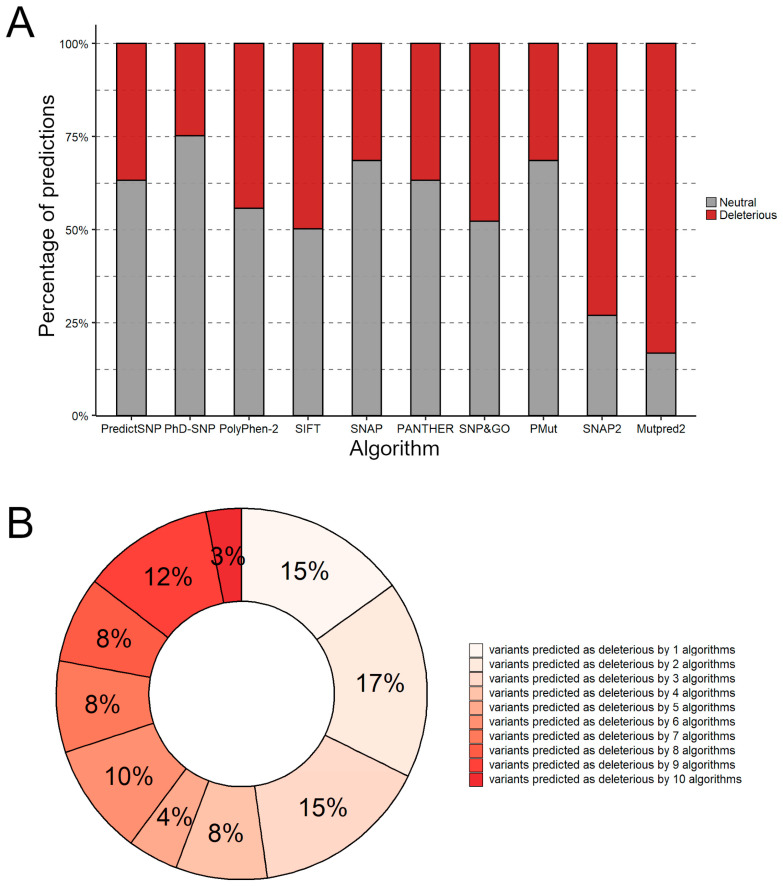
Functional prediction of human FXN protein variants. (**A**) Functional prediction of mutations in each functional prediction algorithm used. The number of mutations predicted to be deleterious is shown in red, while the number of mutations predicted to be neutral is shown in gray. (**B**) Rate of deleterious predictions for the studied mutations.

The SNAP2 and MutPred2 algorithms exhibited a high deleterious prediction rate considering the analysis of all the mutations, with 75% and 85% of variants predicted to belong to this class, respectively (Figure 2A). On the other hand, the PhD-SNP showed a low rate of harmful predictions, classifying only 25% of the mutations as harmful.

Thus, the difference observed between prediction algorithms in classifying FXN mutations highlights the importance of using a combination of algorithms for this analysis. Functional prediction methods use a variety of machine learning algorithms and databases for model training. Furthermore, there is no gold standard method for predicting mutations [18]. Among the most used machine learning algorithms are Support Vector Machine, Decision Tree, Neural Networks [35], and Bayesian methods [36].

These algorithms use Artificial Intelligence concepts, enabling computational learning based on detecting patterns from examples contained in large databases [37]. They were trained on a set of data that serves as an example, where cases are divided into classes associated with a set of variables or attributes, aiming to detect patterns in variables associated with the class in question. A summary detailing the predictive algorithms utilized for characterizing FXN missense mutations is available within Supplementary Materials (File S1). Based on the patterns learned during the training stage, these algorithms can make predictions about new cases with relative accuracy [38].

Thus, a consensus approach was applied to analyze FXN missense mutations. Forty-one percent of the mutations were predicted as deleterious by consensus, that is, by more than half of the algorithms used (≥6 algorithms) (Figure 2B). This finding suggests that these mutations can be detrimental to protein function, especially those predicted as deleterious by all functional prediction algorithms simultaneously since algorithms with different parameters converged on the same result.

Furthermore, the mutations with the highest rates of deleterious predictions were concentrated in the frataxin-like domain, a domain involved in iron metabolism and the synthesis of iron–sulfur clusters. The frataxin-like domain is located between residues 90 and 198, where most of the iron-binding residues and other sites of post-translational modification are found (Figure 1), such as phosphorylation, which is essential for frataxin degradation [27].

**Figure 3:**
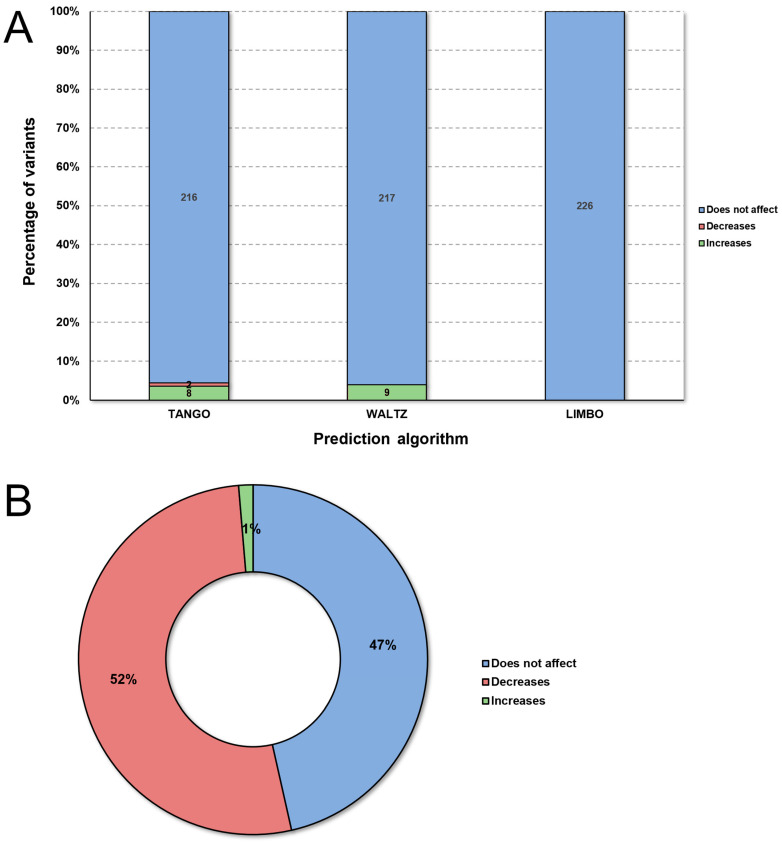
Stability prediction and SNPEffect4.0 analysis of missense mutations in the FXN protein. (**A**) Missense mutations of FXN were compiled and submitted to the WALTZ (amyloid propensity), TANGO (protein aggregation tendency), and LIMBO (chaperone binding) algorithms of SNPEffect4.0. (**B**) The graph shows the percentage of mutations that alter protein stability according to I-Mutant3.0.

Analysis of the SNPeffect, which evaluates protein aggregation tendency, amyloid propensity, and chaperone binding tendency using the TANGO, WALTZ, and LIMBO algorithms (Table S3) [31] (Figure 3A), indicated that nine mutations increased amyloid propensity (WALTZ): V68I, V73A, E108D, D112A, D122Y, L156I, V174A, V174L, and H183R, while eight other mutations were classified as increasing protein aggregation (TANGO) of FXN: A10V, G11V, M76V, E108V, G130A, G130V, D139V, and H177Y. These mechanisms are central to the pathophysiology of neurodegenerative diseases such as Alzheimer’s, Parkinson’s, and Huntington’s diseases. No mutations were predicted to decrease or increase chaperone binding [18,32].

Stability prediction analysis in I-Mutant (Table S3), which applies the “Support Vector Machine” method to calculate free energy based on experimentally determined structures [33] (Figure 3B), suggested that most of the mutations affected protein stability. Fifty-two percent of the mutations were predicted to reduce protein stability, while increased stability was considered a rare phenotype, as only 1% of all the mutations were predicted in this class. Stability changes caused by missense mutations, as observed in more than half of the mutations analyzed, can impact protein function by increasing or decreasing stability.

Stability changes can prevent conformational changes necessary for protein function, including those involved in post-translational modifications and protein–protein interactions [39].

### 2.3. Structural Modeling and Validation

To date, only part of human FXN structure has been experimentally determined by X-ray crystallography [15], which corresponds to the final portion of the protein, spanning amino acids 89–210. Understanding the three-dimensional structures of proteins is necessary, as it improves the understanding of the biological processes that involve the protein in question. This could lead to a better understanding of their activity, their structure–function relationship, and their interaction with other molecules [22]. Therefore, a complete theoretical model of the protein was constructed using in silico modeling.

In silico methods allow the modeling of protein structures efficiently and accurately. Among the available methods, comparative modeling is the most accurate and allows the generation of protein models with a range of general quality that can resemble experimentally determined protein structures [40]. This technique, which is the most dependent on prior information, is based on the use of proteins with potentially related sequences and three-dimensional structures known as models. The experimental structure is selected based on the identity obtained between the target sequence and the possible template candidates available in specific sequence databases [41].

On the other hand, ab initio modeling does not depend on a previously determined model, requiring only information contained in the amino acid sequence of the protein in question. The method uses calculations of physicochemical and thermodynamic properties, based on the global minimum of free energy, aiming to find a conformation with the lowest free energy (global minimum) [42].

Threading modeling, in turn, uses a library of known fragments, from which it identifies the relationships between the structural fragments and the corresponding regions in the target sequence. These fragments are then joined together, generating a temporary three-dimensional structure. Finally, simulations such as Monte Carlo simulations are used to calculate the interaction of these fragments with each other and generate the final model [43].

Among the experimental fragments available in the PDB for human FXN, the 3S4M fragment presented the highest resolution (1.30 Å), coverage (61%), and sequence identity (100%) and was therefore selected for subsequent steps.

During the structural modeling stage, a total of 13 theoretical models of FXN were generated, of which only the models produced by the Rosetta server were completely modeled and folded (Table 1). These models were then used in the subsequent validation steps. The five Robetta models were then aligned in TM-align [44] and showed high structural similarity with the experimental fragment of 3S4M, given their RMSD and TM-score values as shown in Table 2. RMSD and TM-score are structural similarity parameters, where models structurally similar to their templates present RMSD < 2 angstroms and TM-score > 0.5, respectively [22].

The structural quality of the complete models, that is, those generated by the Robetta server, was analyzed using validation algorithms (Table 3).

**Figure 4:**
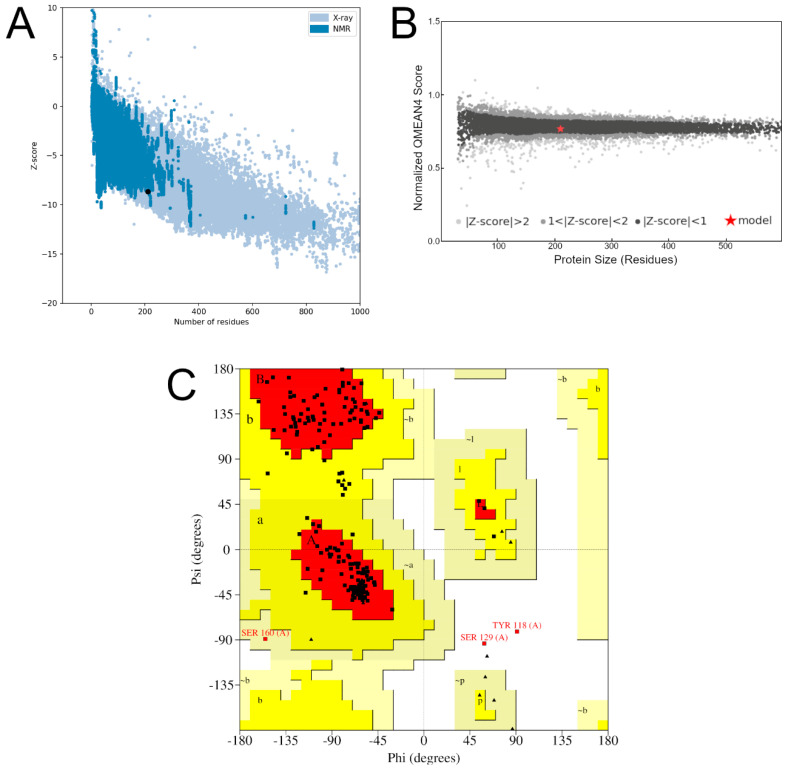
Model validation. (**A**) Validation of model4_robetta on the ProSa-web server. The number of residues is represented on the X-axis and the Z-score of the structure is represented on the Y-axis. The structure submitted to the server corresponds to the black dot. Lighter blue regions correspond to structures already determined by X-ray diffraction, while darker blue regions correspond to structures determined by NMR. (**B**) Validation graph of model4_robetta on the QMEAN server. Structure length in number of residues (X-axis), model QMEAN score (Y-axis). The QMEAN score of the experimental structures is organized into quality bands according to their Z-score, represented in a normal range with a scale that varies from black to gray on the graph. The QMEAN score of the submitted structure is represented by a red “x” in the image. (**C**) Ramachandran graph generated from the validation of model4_robetta on the PROCHECK server. The residues, represented by black squares and triangles, are arranged in favorable regions (red), additional allowed regions (yellow), generously allowed regions (beige), and disallowed regions (white).

ProSa-Web estimates the global quality of a model, i.e., the Z-score, based on the assessment of the potential energy of the three-dimensional structure. ProSa-Web calculates the Z-score of all the structures contained in the Protein Database and plots these values on a graph, which contains the distribution of expected Z-score values for structures determined experimentally by X-ray crystallography and nuclear magnetic resonance (Figure 4A). Finally, the algorithm calculates and includes the Z-score value of the protein of interest in this graph [25].

QMEAN uses six physicochemical descriptors to estimate the overall quality of a given structure (QMEAN score). This QMEAN score is calculated for the target structure and plotted on a graph containing QMEAN score values for 9766 high-resolution protein structures [45] (Figure 4B).

The PROCHECK server, in turn, displays the residuals in a graph called a Ramachandran graph (Figure 4C). In this graph, each protein residue is allocated to different regions based on the arrangement of its phi and psi angles. Structural validation depends on the number of residues allocated to favorable regions of the plot, that is, the regions colored red. The structures that have more than 90% of their waste allocated in these regions are validated by the server [46].

In the Ramachandran plot displayed in Figure 4C, residues Y118 and S129 are located within disallowed regions. Nonetheless, they are neither within nor near the mutation sites at positions 154 and 155 (Figure S1). Consequently, it is unlikely that they significantly influence the microenvironment of the mutated sites. Moreover, to date, there is no experimental evidence suggesting that these residues contribute to protein stability or folding. Therefore, they can be disregarded without compromising the protein structure or the outcomes derived from it, particularly given this study’s focus on the structural effects of I154F and W155R mutations.

**Figure 5:**
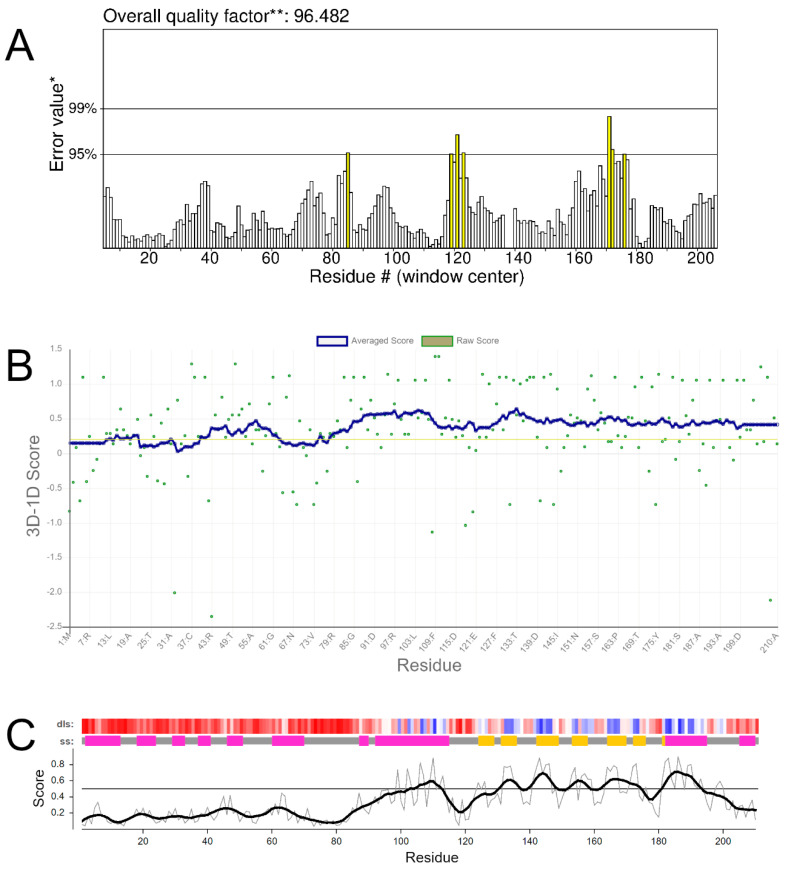
Validation of the generated model. (**A**) Validation graph by the ERRAT algorithm, with residuals plotted on the X-axis and error value on the Y-axis. * On the error axis, two lines are drawn to indicate the confidence with which it is possible to reject regions that exceed that error value. ** Expressed as the percentage of the protein for which the calculated error value falls below the 95% rejection limit. (**B**) Assessment of the structural compatibility of the model on the Verify-3D server. Structure residues are shown on the X-axis, while the 3D-1D score value is on the Y-axis. (**C**) Local and smoothed quality scores calculated for the model on the VoroMQA server. Local scores are shown as thin gray lines, while smoothed scores are depicted with thick black lines. Detailed local structure (dls) and secondary structure (ss) are also presented for a better comparison. Detailed local scores are colored from red to blue, indicating quality from lowest to highest, while secondary structures are color-coded by type: pink for α-helices, yellow for β-sheets, and gray for coils.

ERRAT identifies error-prone regions in protein structures by analyzing the distance of their non-bonded interactions. Based on the observed distribution of these distances, recorded from a dataset containing 96 high-resolution protein structures, the algorithm estimates an error probability for each protein residue. High-resolution structures typically have approximately 95% of their residuals with an error probability of less than 95%. The theoretical model of human frataxin presented 96.48% of its residues within the expected error range, thus being validated [47] (Figure 5A).

The Verify-3D algorithm assigns a structural compatibility value, the 3D-1D score, to each residue and displays the result of this analysis in a graph (Figure 5B). The server uses the minimum value of 0.2 for the 3D-1D score as a cutoff point, considering only residues above this value as validated. High-resolution structures typically have more than 80% of their residues considered approved by the server, that is, ≥ 0.2 of the 3D-1D score [48].

VoroMQA, “Model Quality Assessment based on Voronoi Diagram”, provides an estimate of the overall quality of the structure based on the interactions between atoms in the protein. From these interatomic analyses, the algorithm estimates quality scores per residue, which are plotted on a graph, as seen in Figure 5C. Using the calculated local scores, the algorithm generates an overall quality value for the structure. High-resolution structures typically have global quality scores ≥ 0.4 [49]. The validated frataxin model presented a global quality value of 0.4, thus being validated.

Additionally, considering the structural quality values obtained in the validation algorithms, as well as the respective cutoff points for approval—also presented in Table 3—Model 4 was considered validated. This analysis indicated that Model 4 has comparable quality to experimentally determined structures (ProSa-Web and QMEAN), along with high steric (PROCHECK), geometric (ERRAT), and structural quality to its sequence (Verify-3D).

**Figure 6:**
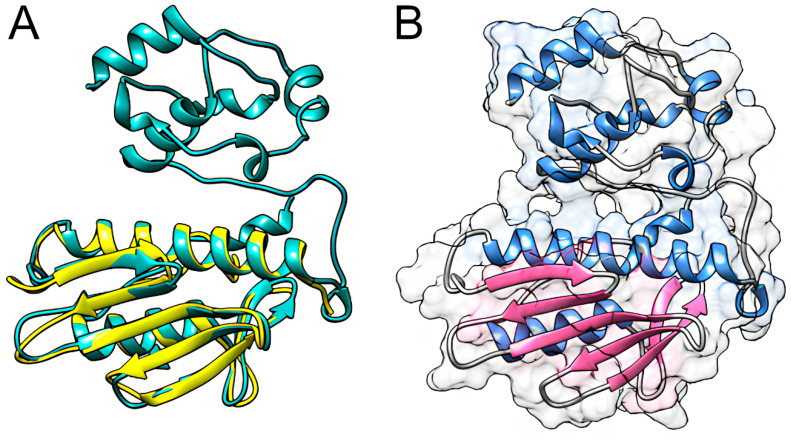
Theoretical model of human FXN generated on the Robetta server. (**A**) Structural alignment in TM-align of Robetta model 4 and experimental fragment 3S4M. The three-dimensional structure of the validated model is represented in green, while the structure of the crystallographic fragment is represented in yellow. (**B**) Final validated model of human FXN. Beta sheet regions are represented by pink arrows, while alpha helix regions are represented by blue helices. The surface of the protein is also shown in the figure.

In Figure 6A, the final validated model is superimposed on the experimental fragment of human frataxin, i.e., 3S4M. Visual inspection of the alignment reaffirms the structural similarity between the validated model and the crystallographic fragment of human FXN, which is required for its structural validation [19]. Noteworthy, the R and R-free values of the template structure (i.e., 3S4M) are 0.153 and 0.187, respectively. It indicates favorable agreement with the experimental data since the R and R-free values are closely matched and approximately 0.20, thus revealing a good fit between the model and the observed structural factors and implying a high level of confidence in the protein structure representation [50,51].

### 2.4. Evolutionary Conservation Analysis

**Figure 7:**
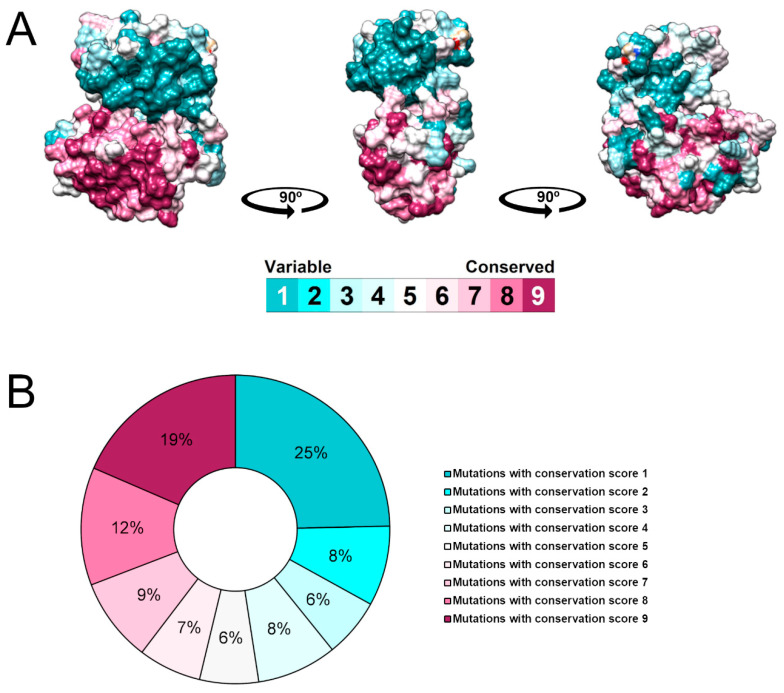
Evolutionary conservation analysis of the human FXN protein using ConSurf. (**A**) The degree of evolutionary conservation of the amino acids of the FXN protein is represented on the protein surface according to the ConSurf color scale, ranging from 1 e cyan (variable) to 9 e brown (conserved). (**B**) Donut chart showing the number of mutations for each degree of evolutionary conservation of the affected position.

The surface of the generated model of the frataxin protein was colored according to the ConSurf color coding scale (Figure 7A), representing the degree of evolutionary conservation of each amino acid in the protein. ConSurf uses a scale that ranges from cyan or 1 (highly variable) to brown or 9 (highly conserved). Amino acid residues with functional or structural importance are crucial for protein function and are often evolutionarily conserved over time due to greater selective pressure [52]. Therefore, the mutations that affect conserved positions are more likely to be harmful to the protein [53].

ConSurf analysis revealed that a substantial portion of the mutations affects conserved (ConSurf score ≥ 7) and variable (ConSurf score ≤ 3) positions within the FXN protein, accounting for 40% and 39%, respectively. Conversely, only 21% of all the mutations occurred in moderately conserved positions (ConSurf score between 4 and 6). Furthermore, 19% of FXN mutations had a ConSurf score of 9, indicating the maximum conservation score and therefore being potentially crucial for FXN function [52].

The frataxin-like domain is a conserved domain found in eukaryotic frataxin and CyaY proteins, the corresponding bacterial orthologs involved in iron–sulfur cluster (FeS) metabolism. Structural analyses of E. coli CyaY, yeast frataxin homolog (Yfh1), and human FXN have revealed a common protein folding pattern across these species [54,55].

A substantial fraction of the mutations compiled in this study for human FXN occurs within the frataxin-like domain (Table S1), from which 36 received the maximum ConSurf conservation score (Table S4). This suggests the functional importance of this domain, housing numerous post-translational modification sites, including iron-binding and phosphorylation sites (Figure 1A).

### 2.5. Molecular Dynamics Simulation

Molecular dynamics (MD) simulation is an in silico method for solving Newtonian equations of motion for a group of atoms, helping to reproduce the behavior of proteins in their biological environment [56]. The atomic coordinates and velocities calculated for the simulated system are recorded over time in the trajectory file, providing detailed information about conformational changes and protein fluctuations over time. The trajectory file is then analyzed to evaluate biochemical and structural parameters, such as structural flexibility [57]. MD simulations can replicate the real behavior of a protein in its natural environment, allowing the study of biomolecular processes such as conformational change, protein folding, and ligand binding, as well as predicting the effects structural perturbations, including mutation, phosphorylation, and protonation [56]. Molecular dynamics simulations were conducted on the wild-type frataxin protein and two missense mutation variants, I154F and W155R. Unlike other FRDA-related mutations in the human FXN protein, the variants I154F and W155R are associated with an earlier onset of the disease and a more severe pathogenesis in affected patients. Given their clinical significance, these mutations have been subjected to further analysis [58,59].

Considering the biochemical characteristics of amino acid substitutions at position I154, replacing isoleucine with another hydrophobic amino acid, such as the bulkier phenylalanine, is unlikely to destabilize protein folding. Particularly considering that this residue is embedded deep within the hydrophobic core of the protein, shielded from the aqueous environment (Figure S1). Despite that, the protein microenvironment may be affected within I154F due to the newly introduced aromatic rings of phenylalanine, which could interact through pi interactions with W173 (Figure S2). In contrast, the surface-exposed and highly conserved residue W155 are more likely to be involved in protein–environment interactions (Figure S1). Additionally, substituting W155 with arginine could lead to repulsive interactions with a nearby arginine residue at position 165 (Figure S2), potentially disrupting the local protein microenvironment [58].

Thus, the mutations I154F and W155R can significantly alter the microenvironment around the substituted residues in the human frataxin protein. These alterations may include the disruption or formation of new interactions as well as changes in local electrostatic and hydrophobic properties [58,60].

In this context, analyzing these mutations through MD simulations provides valuable insights into how changes in the amino acid sequence influence both the local microenvironment and the overall protein structure. While static models like crystallographic structures can be used to identify immediate structural disruptions at mutation sites, MD simulations can capture the dynamic rearrangements that a protein undergoes in response to mutations. Additionally, this method enables a comprehensive evaluation of the cumulative effects of mutations on protein stability and flexibility, extending far beyond the immediate vicinity of the mutated site [32,61].

The root mean square deviation (RMSD) measures the spatial differences between the initial structure and its corresponding coordinates calculated during the simulation. RMSD is used to analyze the atomic displacement between protein structures during an MD simulation and is performed by the molecular dynamics mechanism. This parameter is useful for analyzing the movement of protein structures over time and comparing the convergence of structures during the simulation [62].

**Figure 8:**
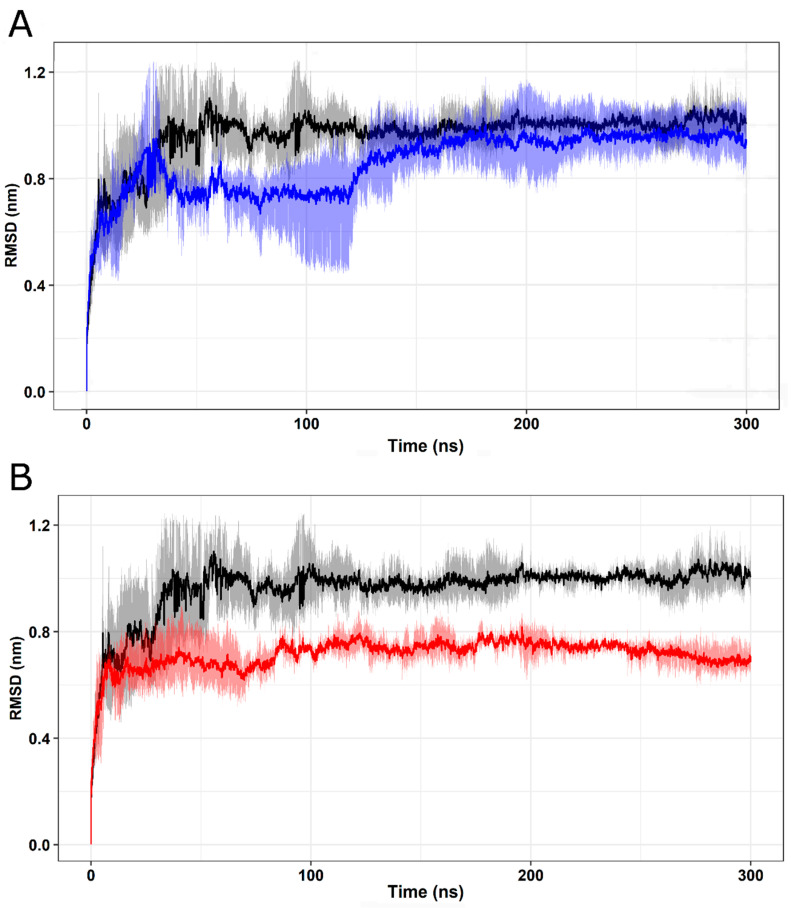
RMSD analysis of native FXN and its variants. The RMSD values calculated for the backbone atoms of the wild-type FXN protein and its variants at 300 K over time. Means (solid lines) and 95% confidence intervals (smooth lines) are shown for triplicates. (**A**) The wild type is represented in black, while the I154F variant is represented in blue. (**B**) The wild type is represented in black, while the W155R variant is represented in red.

RMSD values were calculated from the total number of protein conformations computed in the MD trajectories [62]. As shown in Figure 8, wild-type FXN and I154F (Figure 8A) and W155R (Figure 8B) variants exhibited stable behavior after an initial period of structural instability. After approximately 150 ns, a plateau is observed in the RMSD values, suggesting that these proteins are in a stable average conformation, indicating that the system has reached equilibrium [11,32,53].

**Figure 9:**
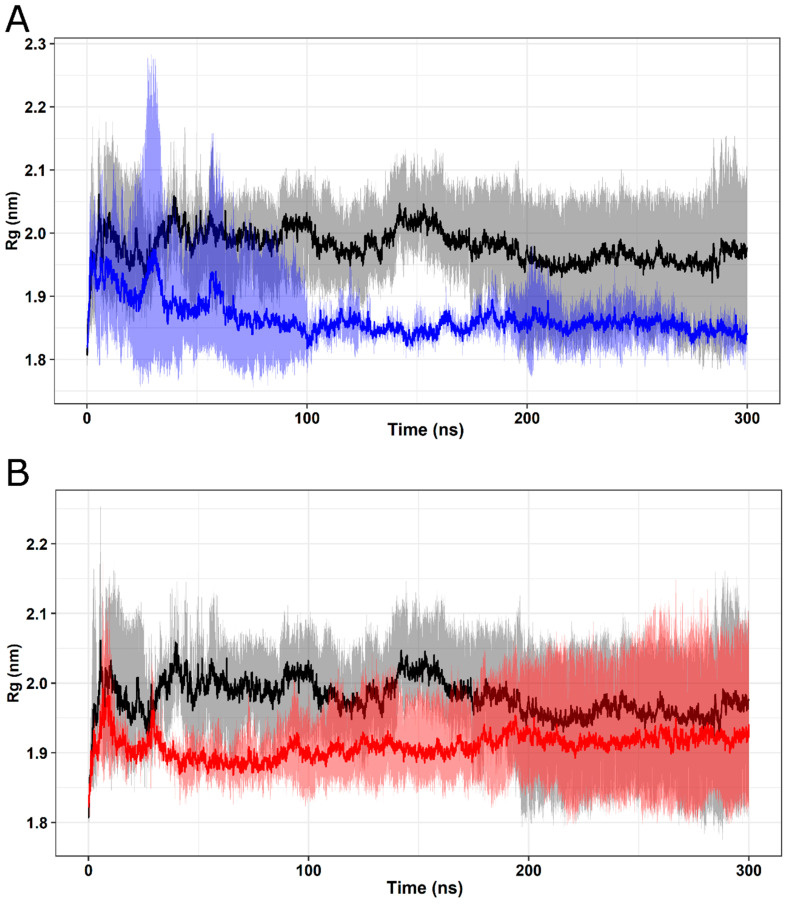
Rg analysis of native FXN and its variants. The Rg values of native FXN and its variants at 300 K are shown over time. Means (solid lines) and 95% confidence intervals (smooth lines) are shown for triplicates. The means of each triplicate are represented by solid lines, while the confidence intervals are represented by smoother lines. (**A**) The wild type is represented in black and the I154F variant in blue. (**B**) The wild type is represented in black and the W155R variant in red.

The radius of gyration (Rg) measures the structural displacement of protein atoms around their common center of mass along the trajectory, providing information about the compaction of the protein over time. The calculated Rg values for wild-type FXN and its variants are depicted in Figure 9. The analyzed protein structures exhibited relatively constant Rg values, indicating stable protein folding [12]. The mean and standard deviation values of Rg for FXN WT (1.917 ± 0.091) are similar to those of the W155R variant (1.968 ± 0.095) (Figure 9B). Meanwhile, the I154F variant (Figure 9A) presented the lowest mean and standard deviation values (1.854 ± 0.032). This result suggests that there were no significant changes in the compaction of the analyzed variants.

**Figure 10:**
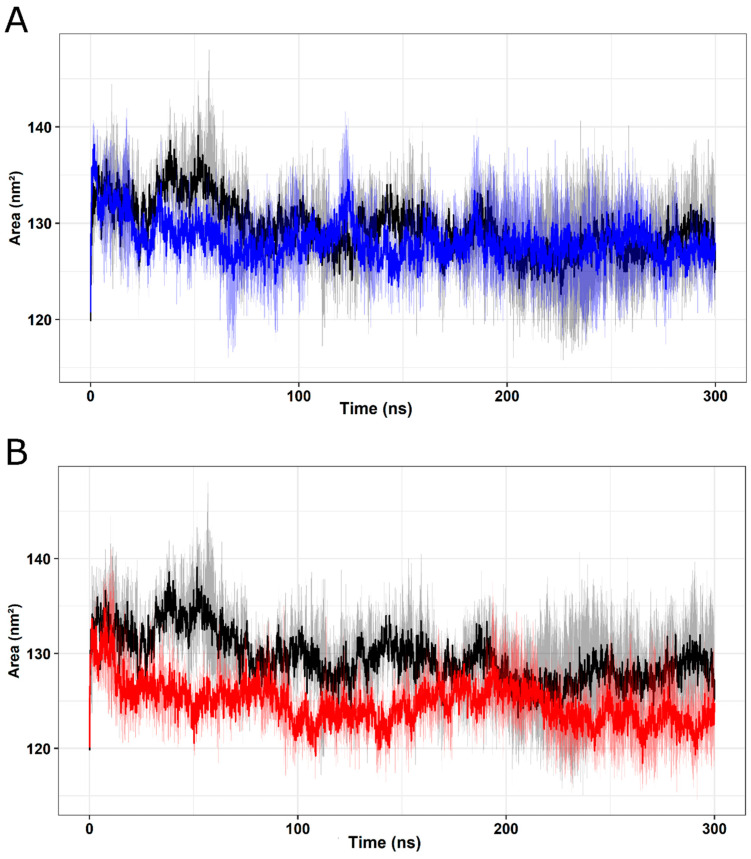
SASA analysis of native FXN and its variants. SASA values of native FXN and its variants up to 300 K are shown over time. Means (solid lines) and 95% confidence intervals (smooth lines) are shown for triplicates. (**A**) The wild type is represented in black and the I154F variant in blue. (**B**) The wild type is represented in black and the W155R variant in red.

SASA measures the exposed surface on a protein structure that can be accessible to solvent molecules, providing crucial information about exposure to proteins over time in their biological environment [63,64]. The SASA values calculated throughout the simulations, represented in Figure 10, indicate stable behavior for the I154F (Figure 10A) and W155R (Figure 10B) variants, showing stable behavior after 150 ns until the end of the MD trajectories. The analysis suggests that the mean SASA values for FXN T (128.0 ± 4.7) are similar to the mean values for the I154F (127.7 ± 3.9) and W155R (124.2 ± 3.4) variants, indicating that there are no significant changes pointing to changes on the protein surface.

**Figure 11:**
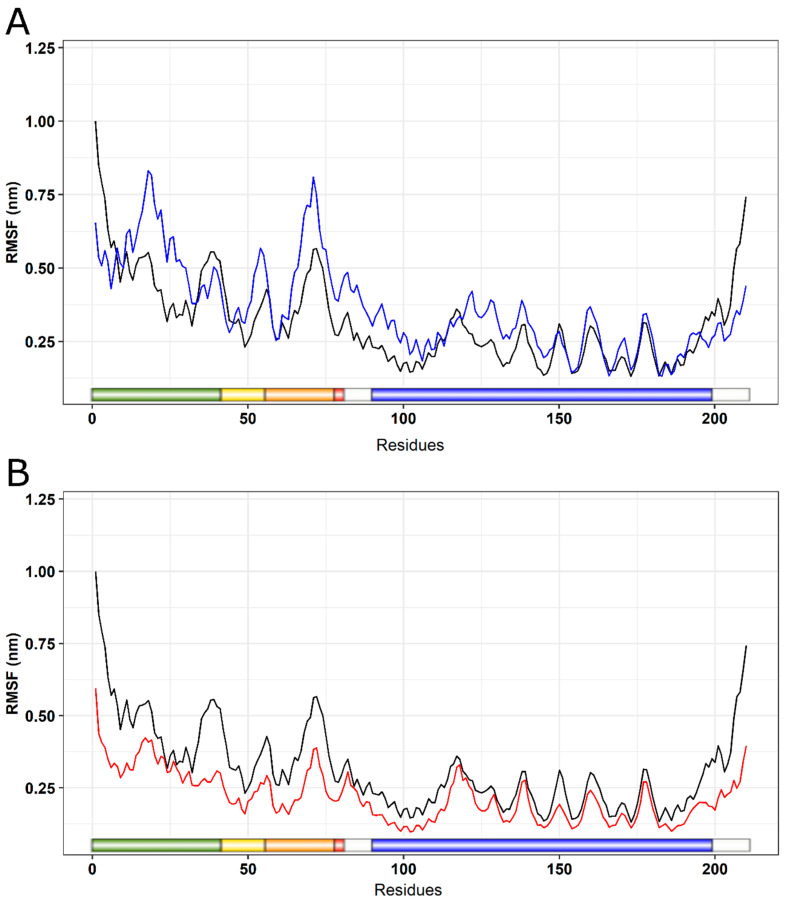
RMSF analysis of native FXN and its variants. The RMSF values for each native FXN residue and its variants are shown in a linear graph at a temperature of 300 K. Shown below is a schematic representation of the structure of the wild-type protein, with key regions highlighted. The transition peptide is colored in green, the frataxin-like domain in blue, and the regions comprising intermediate forms (FXN42, FXN56, and FXN78) are highlighted in yellow, orange, and red, respectively. (**A**) The wild type is represented in black, while the I154F variant is represented in blue. (**B**) The wild type is represented in black, while the W155R variant is represented in red.

RMSF is a parameter used to measure the structural displacement of an amino acid in relation to its average position during the simulation [19]. RMSF evaluates the flexibility of protein structures, leading to the identification of more flexible or rigid regions [65]. An RMSF analysis indicated increased flexibility for the I154F mutation (Figure 11A), with greater changes in the initial domains such as the transition peptide, FXN42, FXN56, FXN78, and mFXN (FXN81). On the other hand, a decrease in flexibility was observed for the W155R variant (Figure 11B).

Consistent with the RMSF analysis, the B factor is used to describe the flexibility of protein residues, being used during the MD simulation to analyze the flexibility of its residues. Analyze the structural displacement of an amino acid with its average position considering the vibrations caused by temperature. Each amino acid of the FXN protein had its value calculated and subsequently projected onto the surface of the protein structure, thus providing a three-dimensional representation of the protein’s structural flexibility [11].

**Figure 12:**
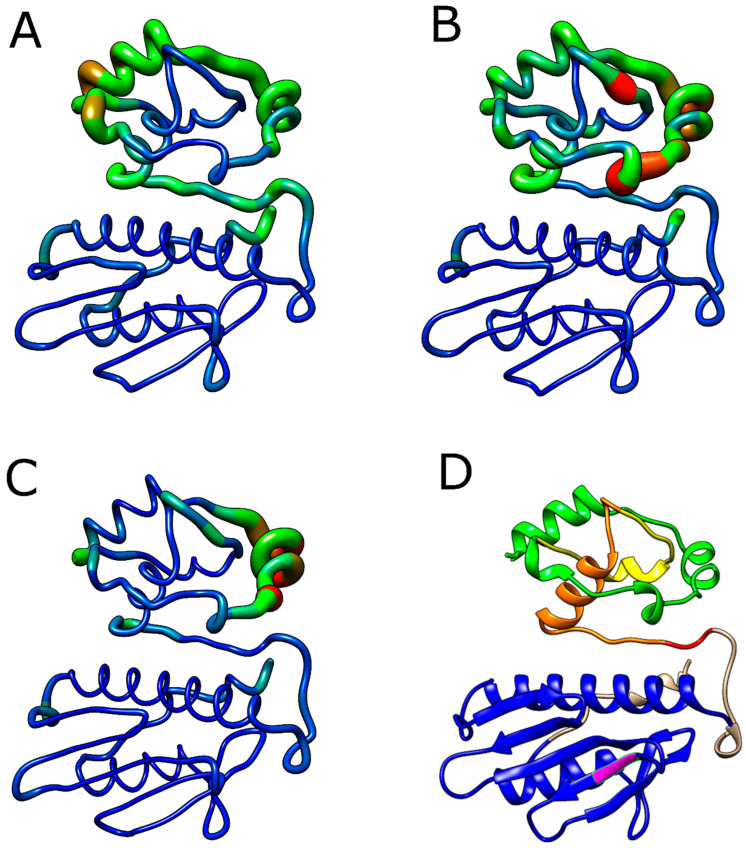
Analysis of native FXN factor B and its variants. Residues of wild-type FXN (**A**) and its variants I154F (**B**) and W155R (**C**) are sized and colored according to their B-factor values, following a color thickness scale ranging from thin blue (residues more rigid) to thick red (more flexible residues). (**D**) Schematic representation of the FXN structure for comparison. The transition peptide is colored green, while the frataxin-like domain is colored blue. The regions comprising intermediate forms (FXN56, FXN78, and FXN81) are highlighted in yellow, orange, and red, respectively. Mutated residues (I154F and W155R) are highlighted in pink.

As shown in Figure 12A, the I154F variant exhibited greater flexibility in the transition peptide and FXN56 regions compared to wild-type FXN. On the other hand, the W155R variant (Figure 12B) showed less flexibility in the same region. Changes in flexibility were observed in both variants in regions similar to those found during the RMSF analysis, suggesting a change in protein flexibility with both variants.

During the protein maturation process, proteins undergo a series of coordinated events, including folding, post-translational modification, and cofactor assembly. These processes are highly dependent on protein flexibility, which allows proteins to acquire their functional three-dimensional structures. Changes in protein flexibility can affect the efficiency and precision of these processes, leading to misfolded or dysfunctional proteins [11].

Essential dynamics (ED), also known as Principal Component Analysis (PCA), is applied to analyze MD simulations. PCA transforms complex, high-dimensional data contained in a molecular trajectory into a low-dimensional space where large-scale protein movements are observed, reducing the number of dimensions needed to describe the dynamics of these proteins. By statistically filtering the observed movements in the molecular trajectory using a covariance matrix with Cartesian coordinates representing the atomic positions of the protein, PCA allows the separation of essential movements of the protein from others. Larger-scale movements, or essential movements, are generally more biologically relevant to protein function, such as opening, closing and bending, while other movements consist of small local fluctuations and are irrelevant [32].

**Figure 13:**
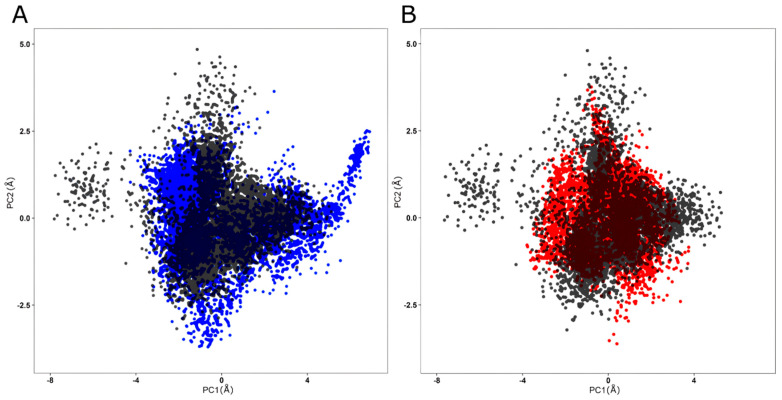
PCA for native FXN and its variants. Projection of the first two principal components extracted from the MD trajectories. (**A**) PCA projection for wild-type FXN in black compared to the I154F variant in blue. (**B**) PCA projection for wild-type FXN in black compared to the W155R variant in red.

The MD trajectories of wild-type FXN and its variants in the subspace covered by PC1 and PC2 are displayed in Figure 13. PCA analysis suggests that the main components, PC1 and PC2, captured the dominant movements of the protein, representing about 89, 74%, 73.31% and 91.39% of the total variance for FXN WT and its variants I154F and W155R, respectively. In Figure 13 it is observed that the variants occupy a similar area in the conformational space, while the I154F variant (Figure 13A) occupies a relatively larger area than the native protein, while the W155R variant (Figure 13B) occupies a smaller area. Changes in the cluster shape, i.e., in the conformational space of the variants, were also observed when compared to the wild-type frataxin cluster. ED analysis therefore indicated changes in the global essential dynamics of all variants.

**Figure 14:**
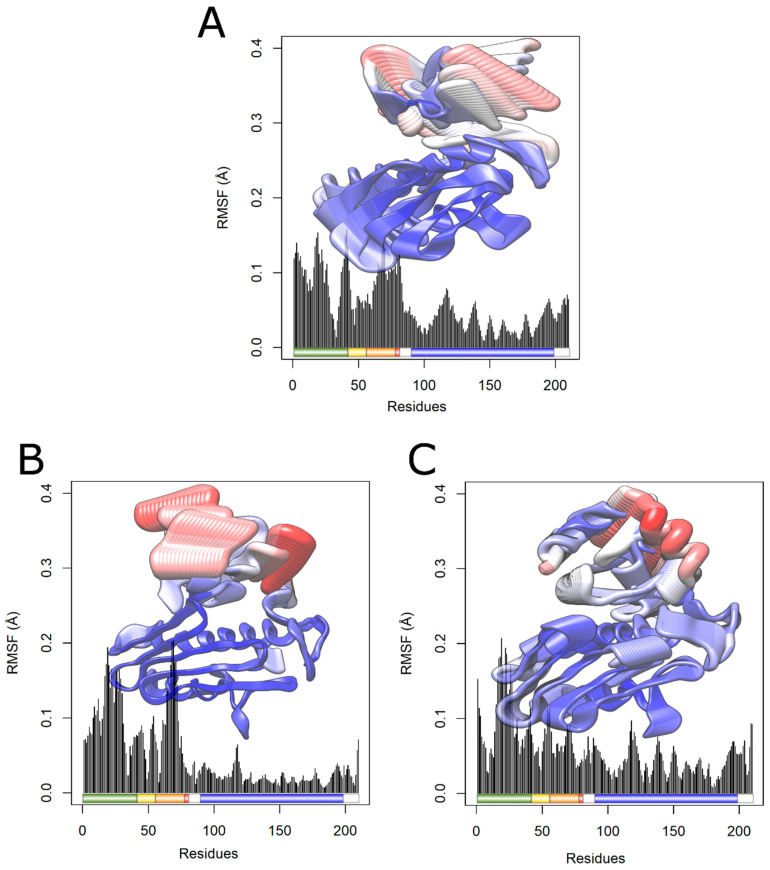
Contribution of RMSF to PC1 of native FXN and its variants. The RMSF contribution of each protein amino acid to PC1 is shown in a linear graph, accompanied by the projection of the corresponding protein structure and schematic representations of key protein regions. The transition peptide is colored green, while the frataxin-like domain is colored blue, and the regions comprising the intermediate forms, FXN56, FXN78, and FXN81, are highlighted in yellow, orange, and red, respectively. Each amino acid, whether from the WT or its two variants under consideration, is colored and scaled according to its RMSF contribution, on a color and thickness scale that ranges from blue and thin (low fluctuations) to red and thick (high fluctuations). (**A**) RMSF contribution of WT FXN to PC1. (**B**) RMSF contribution of Variant I154F to PC1. (**C**) W155R Variant RMSF contribution to PC1.

**Figure 15:**
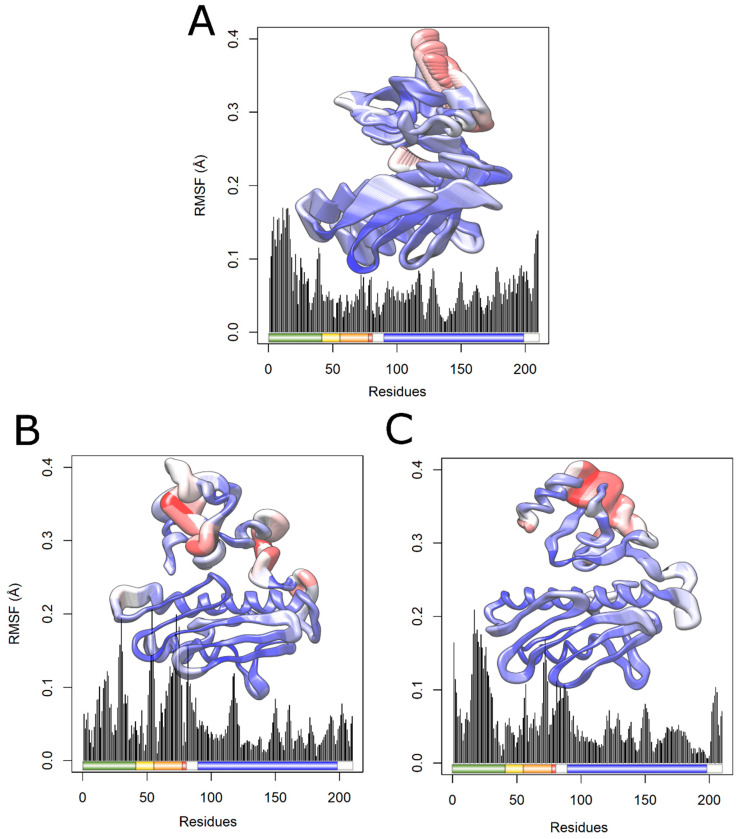
Contribution of RMSF to PC2 of native FXN and its variants. The RMSF contribution of each amino acid of the protein to PC2 is shown in a linear graph, where the corresponding structure is projected and schematic representations of fundamental regions of the protein are highlighted. The transition peptide is colored green, while the frataxin-like domain is colored blue, and the regions that make up the intermediate forms, FXN56, FXN78, and FXN81, have been highlighted in yellow, orange, and red. Each amino acid was colored and sized according to its RMSF contribution, on a color and thickness scale ranging from blue and thin (low fluctuations) to red and thick (high fluctuations). (**A**) Contribution of RMSF to PC2 of FXN WT. (**B**) RMSF contribution to PC2 of variant I154F. (**C**) RMSF contribution to PC2 of the W155R variant.

The contribution of RMSF for each protein amino acid to the main components PC1 and PC2 was also analyzed [32]. The RMSF values for the projections are presented in Figure 14 and Figure 15, respectively. The RMSF for PC1 (Figure 14) showed increased essential mobility in the transition peptide of both variants and in the FXN78 domain of the I154F variant, as well as decreased flexibility in the frataxin-like domain, with greater loss in the I154F variant. On the other hand, the contribution of RMSF to PC2 (Figure 15) showed essential mobility changes in the transition peptide, FXN56, FXN78, and the frataxin-like domain, mainly in the I154F variant. Analyzing Figure 14 and Figure 15, the changes appear to be more pronounced in the transition peptide, in green, and in the FXN56 domain, in orange, with high flexibility fluctuations, mainly in the I154F variant.

To further explore the structural changes induced by mutations, we aligned the structures of variants I154F and W155R with the wild type at the final stage of molecular dynamics (MD) simulations (Figure S3). The structural alignment of these variants with the wild type returned RMSD values of 3.35 and 3.81 Å, suggesting significant structural perturbations that could have functional implications for the protein [23]. These alterations mainly occur within the N-terminal domain of I154F and W155R, supporting the findings from previous analyses of RMSF, B-factor, and PCA.

Human frataxin is a highly conserved, nuclear-encoded protein that requires proteolytic processing to become functional in its mature form. In eukaryotes, frataxin is synthesized as a pre-protein with an N-terminal signal peptide for mitochondrial transport [27,66]. The N-terminal mitochondrial targeting sequence of both mammalian and yeast frataxin undergoes proteolytic cleavage in a two-step process, ultimately yielding the mature protein [27]. Both cleavage steps are mediated by the mitochondrial processing peptidase (MPP) [59,67]. The precursor of yeast frataxin Yfh1p undergoes two sequential cleavages by MPP for maturation [68]. The two-step model is supported by in vitro studies, while in vivo analysis in human cells corroborates this model, revealing a different N-terminal sequence for mature frataxin [27,66]. In vitro self-assembly experiments suggest a critical role for residues 56 to 78 in frataxin polymer formation [69]. Frataxin homopolymerization was analyzed in the Δyfh1 yeast knockout model, showing it is dispensable for rescuing the growth defect but important for reducing iron-induced oxidative stress and increasing life span [70,71].

The impact of FRDA-related mutations in FXN may include effects on folding efficiency, protein stability, proteolytic susceptibility, function, or even protein maturation [67]. According to Gordon et al. (1999), mutations in the N-terminal region can impair signal peptide targeting functions, and mutations in other domains can interfere with the folding necessary to become an efficient substrate for processing [72].

The study of Chi-Lin Tsai et al. (2012) indicated that the I154F and W155R mutations displayed reduced thermodynamic stability in vitro, with a tendency to precipitate after iron binding and highlighted that these mutations impact the maturation and biogenesis of the frataxin protein, associated with the most severe form of FRDA [62]. In vivo assays also conducted by Chi-Lin Tsai et al. (2012) revealed that the FRDA missense mutants W155R and I154F mutants exhibit reduced catalytic efficiency, resulting in an allosteric activation with a weak binding affinity to the Fe-S assembly complex [58].

Moreover, Koutnikova et al. proposed that the interaction and cleavage of frataxin by MPP might be somewhat impaired by disease-associated missense mutations in its C-terminal region (G130V and I154F). They observed that the maturation process of the I154F mutant was diminished in comparison to wild-type frataxin when analyzed in vivo [59].

Proteins are dynamic entities whose functions are intricately tied to their three-dimensional structures and inherent flexibility. Flexibility within a protein structure enables small to large-amplitude domain movements, essential for adapting to optimal conformations necessary for engaging in complex biological processes. Among them, binding to ligands, enzymatic mechanisms, and interactions with other proteins or nucleic acids [73].

Protein recognition is regulated by the interplay between the interaction energy gained from the proper alignment of the ligand in the binding site, and the elastic energy required to deform the interacting molecules. This process is governed by the induced fit model, in which the protein’s binding site is specifically configured to align with a particular ligand, utilizing the protein’s natural flexibility to achieve it. When a protein can adjust its conformation to a ligand, it often leads to more favorable enthalpic interactions, such as hydrogen bonds and van der Waals forces, and may also result in more favorable entropy changes by reducing the conformational freedom of the protein–ligand complex [74].

Protein inherent flexibility, typically maintained in its physiological native state [74], can be disturbed upon mutations and post-translational modifications. These modifications can disturb the balance of forces that maintain protein structure and dynamics, subsequently impacting the protein’s ability to assume an optimal and, thus, functional conformation. While increased flexibility can enhance conformational adaptability, benefiting molecular recognition and target binding, excessive flexibility may lead to structural instability and misfunction. Decreased flexibility, on the other hand, are likely to hinder the protein’s adaptability, potentially leading to loss of function. Nevertheless, the consequences of these alterations can vary significantly, depending on the specific protein and the biological context and environment in which it operates. Thus, the general understanding is that protein flexibility can have strong and yet non-intuitive consequences for protein interaction profile, as reviewed elsewhere [75].

In our study, we analyzed the RMSF and B factor of the wild-type frataxin protein and its I154F and W155R variants. Our findings showed significant changes in frataxin’s flexibility, particularly in the N-terminal region, with a more pronounced effect observed in the I154F variant. The affected regions included the transition peptide domains (FXN1-41), FXN56, and FXN78, crucial for protein maturation and cleavage processes [27].

Thus, our MD findings indicate that the mutations I154F and W155R disturb FXN’s inherent structure and dynamics, primarily in the N-terminal domain. It could compromise the protein’s ability to achieve an optimal and, consequently, functional conformation with a consequent impact on FXN recognition and target binding, especially within the most affected region [75]. Given the key role of the N-terminal domain in protein maturation through interactions with MPP, these mutations may hinder FXN’s recognition, thus impacting the cleavage steps involved in its maturation, aligning with the findings of the previous literature [58,59,72,76,77,78].

## 3. Materials and Methods

### 3.1. Protein Sequence and Variant Acquisition

The sequence of the wild-type human FXN protein was retrieved from the UniProt database (ID: Q16595). Genetic variants of the FXN protein were collected from multiple sources, including the following databases: UniProt (ID: Q16595) [79], OMIM (ID: 606829) [80], dbSNP [34], as well as through a literature review on PubMed.

### 3.2. Predictive Analysis

The native protein sequence and the compiled variants were subjected to functional and stability prediction analyses. Ten algorithms were used to predict the functional impact of FXN variants: PolyPhen-2, SNAP2, SNP&GO, PANTHER, SIFT, SNAP, PHD-SNP, PMut, PREDICT-SNP, and MutPred2. The I-Mutant algorithm was used to evaluate the effect of variants on protein stability. Furthermore, SNPEffect4.0’s TANGO, WALTZ, and LIMBO algorithms were used to predict the potential effects of the mutations on protein aggregation, amyloid propensity, and chaperone binding tendency, respectively [11,18].

### 3.3. Structural Modeling and Validation

The experimental fragment of the human FXN protein was retrieved from the Protein Data Bank (PDB) (3S4M) [15], selected based on the highest coverage and sequence identity determined through a Protein Blast search algorithm. Furthermore, the quality of resolution was considered when selecting the structure, which served as a model for comparative modeling. Comprehensive theoretical models of the native protein were constructed through comparative modeling, threading and/or ab initio methods using the following modeling servers: SwissModel, Robetta, I-TASSER, MholLine, and Raptor-X [17,19]. Models exhibiting incomplete sequence coverage or folding were excluded from further analysis.

The structural quality of the complete theoretical models of human FXN was assessed using six validation algorithms: ProSa-Web, QMEAN, PROCHECK, Verify3D, ERRAT, and VoroMQA. These algorithms evaluate structure–sequence compatibility, evaluate overall model quality via comparisons with experimentally determined structures, and perform stereochemical analyzes using the Ramachandran plot [17]. The theoretical model that presented the highest number of approvals in the validation algorithms was chosen for the structural alignment stage.

Finally, the selected model was structurally aligned with the 3S4M experimental fragment using the TM-align server to verify its similarity with the crystallographic structure of the human FXN protein [44].

### 3.4. Evolutionary Conservation Analysis

The validated model was subjected to an evolutionary conservation analysis on the ConSurf server [81], which calculates the degree of evolutionary conservation of each amino acid in the protein based on the phylogenetic relationship between the target protein and its homologous sequences through multiple sequence alignments [11]. The following parameters were selected for analysis: homologous search algorithm: PSI-BLAST; number of iterations: 3; E cut-off value: 0.0001; protein database: UniProt; reference sequence: closest; number of selected reference sequences: 150; maximum sequence identity: 95%; minimum identity of counterparties: 35%; alignment method: MAFFT-L-INS-I; calculation method: Bayesian; evolutionary replacement model: best model (default) [17].

### 3.5. Molecular Dynamics Simulation

The I154F and W155R mutations were individually induced via in silico mutagenesis in the validated native FXN model using the Mutator Plugin 1.3 [82] within the Visual Molecular Dynamics (VMD) 1.9.3 software [83]. Molecular dynamics (MD) simulations of the native protein and variants were carried out using the GROMACS 2018.3 package [84], following methodologies established by our group [11,12]. The simulations were carried out in triplicate using the AMBER99SB-ILDN force field, known for accurately describing several structural and dynamic properties of proteins [85].

Initially, the molecules were solvated in a triclinic box and solvated with TIP3P water molecules. The system was neutralized by adding Na+ and Cl- ions at a concentration of 0.15 mol/L. Subsequently, energy minimization was performed using the steepest descent method [86,87]. The minimized system experienced NPT (constant number of particles, constant pressure, and temperature) and NVT (constant number, volume, and temperature) sets for 100 ps under a constant pressure of 1 atm and a temperature of 300 K. Following the NPT simulation, MD simulations were performed in triplicate for 300 ns under a temperature of 300 K.

The MD trajectories were comparatively analyzed using several GROMACS distribution programs: *gmx trjcat*, *gmx trjconv*, *gmx rms*, *gmx rmsf*, *gmx gyrate*, *gmx sasa*, *gmx hbond*, *gmx mindist*, *gmx distance*, and *gmx gangle*. Parameters including root mean square deviation (RMSD), root mean square fluctuation (RMSF), radius of gyration (Rg), number of intramolecular hydrogen bonds, solvent accessible surface area (SASA), and B factor were examined. Furthermore, principal component analysis (PCA) was performed for native FXN and its variants. Rotational and translational motions were eliminated to construct covariance matrices, and PCA was performed on the Cα atoms. The Cartesian coordinates of these atoms were used to generate the covariance matrices. PCA analyzes were performed using the Bio3D package of the R software version 4.3.3 (https://www.r-project.org/ accessed on 3 April 2024) [88]. Data visualization was performed using the *ggplot2* package [89] in R and the UCSF Chimera software version 1.14 (https://www.cgl.ucsf.edu/chimera/ accessed on 2 February 2024) [90]. The structures of wild type and variants at the final stage of molecular dynamics (MD) simulations were aligned in the TM-align server [91] to further explore the structural changes induced by the mutations I154F and W155R.

## 4. Conclusions

Overall, 226 missense mutations in human frataxin were compiled from the literature and databases, which underwent a thoroughly functional characterization in silico. This study also provided an unprecedented, complete, and accurate three-dimensional model of human frataxin, serving as a basis for constructing the structure of clinically relevant variants, I154F and W155R. Our MD findings suggest that these mutations disturb FXN’s inherent structure and dynamics, primarily within the N-terminal domain. This behavior could compromise the protein’s ability to adopt functional conformations, potentially leading to impaired recognition and cleavage by the MPP protein, which is directly involved in FXN maturation, as outlined in previous studies. Thus, our findings provide valuable insights into the molecular basis of FXN dysfunction in FRDA, shedding light on future directions that could be explored for developing new therapeutic strategies.

## Figures and Tables

**Table 1:** Structural modeling of the human frataxin protein.

Algorithm	Model	Model Size	Folding
Rosetta	1	210	complete
Rosetta	2	210	complete
Rosetta	3	210	complete
Rosetta	4	210	complete
Rosetta	5	210	complete
I-Tasser *	1	210	incomplete
I-Tasser *	2	210	incomplete
I-Tasser *	3	210	incomplete
I-Tasser *	4	210	incomplete
I-Tasser *	5	210	incomplete
Raptor-X *	1	210	incomplete
MholLine *	1	210	incomplete
Swiss Model *	1	168	complete

* Models with incomplete size and/or folding.

**Table 2:** RMSD and TM-Score values calculated from the structural alignment with the 3S4M fragment.

Algorithm	Model	RMSD	TM Score
Rosetta	1	0.59	0.98055
Rosetta	2	0.67	0.97807
Rosetta	3	0.59	0.98093
Rosetta	4	1.53	0.90771
Rosetta	5	0.70	0.97564

**Table 3:** Structural validation for the complete theoretical models of the human frataxin protein.

Model	ERRAT ^1^	PROCHECK ^2^	Verify-3D ^3^	Prosa-Web ^4^	QMEAN ^5^	VoroMQA ^6^
Rosetta1	99	86	80	NMR	high resolution	0.43
Rosetta2	99	84	77	NMR	high resolution	0.43
Rosetta3	95	87	86	NMR	high resolution	0.42
Rosetta4	96	91	80	NMR	high resolution	0.40
Rosetta5	98	86	83	NMR	high resolution	0.42

^1^ Overall quality index in ERRAT (%); ^2^ Percentage of waste in the most favorable regions of the Ramachandran plot; ^3^ Percentage of waste with a 3D-1D compatibility score equal to or greater than 0.2; ^4^ The overall quality index (Z-score) estimated for the model is within the range of Z-score values calculated for NMR or crystallographic structures; ^5^ The QMEAN score estimated for the model is within the range of QMEAN score values calculated for high-resolution protein structures; ^6^ VoroMQA Global Quality Index.

## Data Availability

All relevant data are within the manuscript and Supplementary Materials.

## Supplementary Materials

The following supporting information can be downloaded at: https://www.mdpi.com/article/10.3390/ijms25115796/s1. References [92,93,94,95,96,97] are cited in the Supplementary Materials in the SI section.
