# Supplementary material for: An In Silico Analysis of Genetic Variants and Structural Modeling of the Human Frataxin Protein in Friedreich’s Ataxia
Source: Int J Mol Sci. 2024 May 26;25(11):5796. doi: 10.3390/ijms25115796 (PMC11172458; doi:10.3390/ijms25115796)
Supplement: Supplementary file 1 [file ijms-25-05796-s001.zip › File S1.pdf]

File S1. Detailed Description of the Predictive Algorithms used for Characterizing FXN missense mutations.

The PMut algorithm is trained on a dataset extracted from the SwissProt database using Neural Networks as a learning method to analyze parameters such as evolutionary conservation of the substituted amino acid residue, protein information and physicochemical properties [2]. On the other hand, the SNAP algorithm uses Neural Networks to perform functional analyzes from the Mutant Protein Database, using parameters for analysis derived from the amino acid sequence of the protein, evolutionary and structural aspects of the protein such as solvent accessibility and secondary structure [3].

The SNPs&GO algorithm uses Support Vector Machine to make predictions, based on information from a dataset containing neutral and deleterious SwissVar variants. In this sense, the algorithm analyzes the functionality and domain affected by the mutation, changes in the secondary structure and evolutionary conservation of the residue in question [4]. The SIFT algorithm relies on multiple sequence alignment (PSI-blast) and probability matrix, using the dbSNP database as a basis to analyze evolutionary characteristics of the sequence in question before carrying out its predictive analysis [5]. Meanwhile, PolyPhen-2 algorithm uses Naïve classifier based method Bayes and trains on databases such as HumDiv and HumVar , relying on evolutionary and structural features such as electrostatic charge, secondary structure, hydrophobicity, solvent accessibility, number of interactions, surface area, and side chain volume to perform its analysis [6].

The PhD-SNP algorithm uses the Support Vector Machine method, trained on a dataset derived from SwissProt, to analyze the evolutionary conservation of the amino acid substituted in the mutation [7]. MutPred2 uses the neural network-based method, relying on a dataset derived from the HGMD, SwissVar and dbSNP databases. Evaluates solvent-accessible surface, secondary structure, metallic bonding and post- translational modifications to perform predictive analyzes [8]. The SNAP2 algorithm also uses the Neural Networks-based method, trained on a dataset derived from the Mutant Protein Database. It analyzes parameters such as information about the domain affected by the mutation, evolutionary conservation, secondary structure, solvent accessibility and flexibility [3].

Panther uses the hidden Markov model, functioning as a statistical model where a system is modeled with unknown parameters based on observable parameters. Trained on the human mutation database, it analyzes the functionality of the mutation and the evolutionary conservation of the residue [9]. Finally, the Predict -SNP algorithm use a consensus methodology, using a dataset derived from the UniProtKB and Protein Mutant databases, where it analyzes scores from eight different algorithms, such as MAPP, nsSNPnalyser, Panther, SIFT, SNAP, PolyPhen- 1, PolyPhen-2 and dbSNP [10].

References

1. Batista GEAPA, Monard MC. An analysis of four missing data treatment methods for supervised learning. Appl Artif Intell. 2003;17: 519–533. doi:10.1080/713827181
2. López-Ferrando V, Gazzo A, De La Cruz X, Orozco M, Gelpí JL. PMut: A web-based tool for the annotation of pathological variants on proteins, 2017 update. Nucleic Acids Res. 2017;45: W222–W228. doi:10.1093/nar/gkx313
3. Bromberg Y, Rost B. SNAP: Predict effect of non-synonymous polymorphisms on function. Nucleic Acids Res. 2007;35: 3823–3835. doi:10.1093/nar/gkm238
4. Capriotti E, Calabrese R, Fariselli P, Martelli PL, Altman RB, Casadio R. WS-SNPs&GO: a web server for predicting the deleterious effect of human protein variants using functional annotation. BMC Genomics. 2013;14 Suppl 3. doi:10.1186/1471-2164-14-s3-s6
5. Kumar P, Henikoff S, Ng PC. Predicting the effects of coding non-synonymous variants on protein function using the SIFT algorithm. Nat Protoc. 2009;4: 1073–1081. doi:10.1038/nprot.2009.86
6. Adzhubei I, Jordan DM, Sunyaev SR. Predicting functional effect of human missense mutations using PolyPhen-2. Curr Protoc Hum Genet. 2013;Chapter 7: Unit7.20. doi:10.1002/0471142905.hg0720s76
7. Capriotti E, Calabrese R, Casadio R. Predicting the insurgence of human genetic diseases associated to single point protein mutations with support vector machines and evolutionary information. Bioinformatics. 2006;22: 2729–2734. doi:10.1093/bioinformatics/btl423
8. Pejaver V, Urresti J, Lugo-Martinez J, Pagel KA, Lin GN, Nam HJ, et al. Inferring the molecular and phenotypic impact of amino acid variants with MutPred2. Nat Commun. 2020;11. doi:10.1038/s41467-020-19669-x
9. Shan Y, Guan D, Liu J, Mi Z, Liu Z, Liu J, et al. Methodology and applications of city level CO2 emission accounts in China. J Clean Prod. 2017;161: 1215–1225. doi:10.1016/j.jclepro.2017.06.075
10. Bendl J, Stourac J, Salanda O, Pavelka A, Wieben ED, Zendulka J, et al. PredictSNP: Robust and Accurate Consensus Classifier for Prediction of Disease-Related Mutations. PLoS Comput Biol. 2014;10: 1–12. doi:10.1371/journal.pcbi.1003440
