# Supplementary material for: An In Silico Analysis of Genetic Variants and Structural Modeling of the Human Frataxin Protein in Friedreich’s Ataxia
Source: Int J Mol Sci. 2024 May 26;25(11):5796. doi: 10.3390/ijms25115796 (PMC11172458; doi:10.3390/ijms25115796)
Supplement: Supplementary file 1 [file ijms-25-05796-s001.zip › Table S1.pdf]

Table S1. Frataxin protein variants compiled in the dbSNP database.

Variants	rsID	Clinical significance
M1V	rs147643987	Unknown
M1L	rs147643987	Unknown
M1I	rs104894108	Deleterious
M1K	rs142133355	Unknown
M1T	rs142133355	Unknown
W2L	rs1831535584	Unknown
L4P	rs1197008330	Unknown
G5R	rs960895342	Unknown
R6L	rs992322273	Unknown
R6H	rs992322273	Unknown
R7H	rs1194735245	Unknown
A8G	rs775586060	Unknown
A8T	rs1831536106	Unknown
V9I	rs1431787480	Unknown
A10T	rs947792827	Unknown
A10V	rs1831536332	Unknown
G11V	rs1373837878	Unknown
G11S	rs1639970994	Unknown
L12I	rs1168839252	Unknown
L12V	rs1168839252	Unknown
L13Q	rs1462047732	Unknown
A14E	rs761236678	Neutral
P16H	rs1384835969	Unknown
P16S	rs1392880712	Unknown
S17T	rs1831536874	Unknown
P18A	rs1275914423	Unknown
P18T	rs1275914423	Unknown
A19S	rs1316684728	Unknown
A21V	rs1831537475	Unknown
A21D	rs1831537475	Unknown
Q22R	rs1831537597	Unknown
T23I	rs572324212	Unknown
T23N	rs572324212	Unknown
T23A	rs777206398	Unknown
T23S	rs572324212	Unknown
L24F	rs1052328470	Unknown
T25N	rs1423258307	Unknown
T25A	rs1831537991	Unknown
R26W	rs536676174	Unknown
R26Q	rs1167243329	Unknown
V27F	rs1831538235	Unknown
P28R	rs1292894778	Unknown
R29Q	rs1385313783	Unknown
R29L	rs1385313783	Unknown
P30L	rs1339514881	Unknown
P30Q	rs1339514881	Unknown

P30T	rs1295948276	Unknown
E32K	rs555289797	Unknown
E32Q	rs555289797	Unknown
L33V	rs767135118	Neutral
P35L	rs1244615817	Unknown
C37R	rs1261839617	Unknown
G38S	rs576852565	Unknown
G38C	rs576852565	Unknown
R39L	rs1564326540	Unknown
R39H	rs1564326540	Unknown
R39C	rs1010056458	Unknown
R40C	rs145854903	Deleterious
G41V	rs1831539533	Unknown
G41R	rs1388134271	Unknown
L42R	rs1028088816	Unknown
R43H	rs752207437	Unknown
R43P	rs752207437	Unknown
R43L	rs752207437	Unknown
R43C	rs1486151799	Unknown
T44N	rs544129099	Neutral
T44I	rs544129099	Neutral
T44P	rs1423319770	Unknown
D45N	rs755663313	Unknown
D45H	rs755663313	Unknown
I46N	rs1464752486	Unknown
I46S	rs1464752486	Unknown
I46T	rs1464752486	Unknown
I46V	rs1831540145	Unknown
I46M	rs565294240	Unknown
D47E	rs1202974994	Unknown
A48T	rs1358112208	Unknown
A48V	rs1156655103	Unknown
T49N	rs995690945	Unknown
T49P	rs1587811863	Unknown
T49S	rs995690945	Unknown
C50R	rs1304232460	Unknown
C50Y	rs1376272802	Unknown
C50F	rs1376272802	Unknown
T51M	rs1395007559	Unknown
T51A	rs1831540731	Unknown
P52S	rs992332123	Unknown
P52T	rs992332123	Unknown
P52L	rs1026671871	Unknown
R53H	rs1023779193	Unknown
R53L	rs1023779193	Unknown
R54H	rs979225932	Unknown
R54C	rs969560796	Unknown
R54G	rs969560796	Unknown
A55T	rs1316008371	Unknown

S56N	rs1831753352	Unknown
S56R	rs1831753436	Unknown
S57L	rs180718803	Unknown
Q59H	rs1273492939	Unknown
R60H	rs141858334	Neutral
R60C	rs373519193	Unknown
G61V	rs902854618	Unknown
G61S	rs1239150831	Unknown
L62P	rs763682148	Unknown
N67S	rs146284289	Unknown
V68A	rs778674959	Unknown
V68G	rs778674959	Unknown
V68I	rs1223712761	Unknown
V73A	rs1051718716	Unknown
Y74C	rs750274023	Unknown
M76V	rs59907886	Neutral
M76T	rs1273772459	Unknown
K80N	rs1169387979	Unknown
S81T	rs555673369	Unknown
L84W	rs57777433	Unknown
L84F	rs747283339	Unknown
G85A	rs57215370	Unknown
G85V	rs57215370	Unknown
H86P	rs1587818053	Unknown
G88C	rs1340263641	Unknown
D91H	rs373668521	Unknown
E92K	rs1831884210	Unknown
T93I	rs778620538	Unknown
T94A	rs766667087	Unknown
Y95H	rs1258217167	Unknown
Y95C	rs1184832782	Unknown
E96K	rs762990650	Unknown
R97K	rs1378785673	Unknown
R97G	rs1181092877	Unknown
A99T	rs766086086	Unknown
E100A	rs541981554	Unknown
T102M	rs964046026	Unknown
D104E	rs1831885244	Unknown
S105F	rs267602259	Unknown
L106S	rs104894105	Deleterious
L106V	rs1285151894	Unknown
A107P	rs1831885649	Unknown
E108V	rs1587821826	Unknown
E108D	rs1564333434	Unknown
F110S	rs1831885938	Unknown
D112H	rs1239243428	Unknown
D112Y	rs1239243428	Unknown
D112A	rs1587821839	Unknown
L113I	rs1219540200	Unknown

A114V	rs767712381	Unknown
D115E	rs756215781	Unknown
K116E	rs1165716226	Unknown
P117T	rs867960097	Unknown
P117L	rs778049381	Unknown
Y118C	rs749780215	Unknown
T119K	rs757847415	Unknown
T119M	rs757847415	Unknown
D122Y	rs142157346	Unknown
Y123F	rs1831886976	Unknown
G130A	rs104894107	Deleterious
G130V	rs104894107	Deleterious
G130S	rs1681448766	Unknown
T133A	rs1340783297	Unknown
V134I	rs759441343	Unknown
V134G	rs1316786478	Unknown
K135R	rs771963647	Unknown
G138R	rs775491351	Unknown
D139Y	rs760965156	Unknown
D139V	rs764352477	Unknown
N146K	rs146818694	Deleterious
Q148R	rs140472905	Unknown
T149M	rs551991546	Unknown
T149A	rs1424491000	Unknown
K152E	rs1432630081	Unknown
Q153R	rs77781994	Unknown
Q153H	rs780387020	Unknown
I154V	rs104894106	Deleterious
I154F	rs104894106	Deleterious
W155R	rs138471431	Deleterious
L156I	rs1423162963	Unknown
L156P	rs143340609	Unknown
S158A	rs748916855	Unknown
S158P	rs748916855	Unknown
S160C	rs1372039162	Unknown
S161T	rs1832143962	Unknown
S161R	rs1475266569	Unknown
K164R	rs1832295048	Unknown
R165H	rs143396368	Unknown
R165C	rs138034837	Unknown
R165P	rs143396368	Unknown
Y166F	rs901095040	Unknown
G170W	rs1345161827	Unknown
K171E	rs1280128357	Unknown
K171R	rs781204747	Unknown
W173G	rs56214919	Deleterious
V174L	rs1832295554	Unknown
V174A	rs1212674570	Unknown
Y175F	rs1052201	Unknown

H177Y	rs747833464	Unknown
D178E	rs61754561	Neutral
D178N	rs777926714	Unknown
G179S	rs1189451813	Unknown
V180M	rs774482038	Unknown
S181C	rs568327835	Unknown
L182F	rs139616452	Unknown
L182H	rs149335881	Unknown
H183Y	rs1228124556	Unknown
H183L	rs144610605	Unknown
H183R	rs144610605	Unknown
L186R	rs148443992	Unknown
A187V	rs1474150285	Unknown
A188T	rs776068810	Unknown
T191S	rs200532520	Unknown
T191A	rs200532520	Unknown
A193P	rs762731436	Unknown
T196A	rs765980325	Unknown
K197R	rs1832296959	Unknown
L198V	rs1036228270	Unknown
L198R	rs144104124	Unknown
D199N	rs751170542	Unknown
D199G	rs754523390	Unknown
L200S	rs1379863332	Unknown
S202C	rs1052195	Unknown
S202P	rs1384605005	Unknown
L203F	rs1832297474	Unknown
A204P	rs780954419	Unknown
S206Y	rs1289276430	Unknown
S206C	rs1289276430	Unknown
S206T	rs1832297570	Unknown
G207R	rs777584849	Unknown
D209G	rs74621026	Neutral
