# Supplementary material for: An In Silico Analysis of Genetic Variants and Structural Modeling of the Human Frataxin Protein in Friedreich’s Ataxia
Source: Int J Mol Sci. 2024 May 26;25(11):5796. doi: 10.3390/ijms25115796 (PMC11172458; doi:10.3390/ijms25115796)
Supplement: Supplementary file 1 [file ijms-25-05796-s001.zip › Table S3.pdf]

Table S3. Analysis of frataxin protein variants according to I-Mutant3.0 and SNPeffect.

Variants	I-MUTANT3.0	TANGO	WALTZ	LIMBO
M1L	Does not affect	Does not affect	Does not affect	Does not affect
M1K	Decreases	Does not affect	Does not affect	Does not affect
M1T	Decreases	Does not affect	Does not affect	Does not affect
M1V	Decreases	Does not affect	Does not affect	Does not affect
M1I	Increases	Does not affect	Does not affect	Does not affect
W2L	Does not affect	Does not affect	Does not affect	Does not affect
L4P	Decreases	Does not affect	Does not affect	Does not affect
G5R	Does not affect	Does not affect	Does not affect	Does not affect
R6H	Decreases	Does not affect	Does not affect	Does not affect
R6L	Does not affect	Does not affect	Does not affect	Does not affect
R7H	Decreases	Does not affect	Does not affect	Does not affect
A8G	Decreases	Does not affect	Does not affect	Does not affect
A8T	Decreases	Does not affect	Does not affect	Does not affect
V9I	Decreases	Does not affect	Does not affect	Does not affect
A10T	Decreases	Does not affect	Does not affect	Does not affect
A10V	Does not affect	Increases	Does not affect	Does not affect
G11S	Decreases	Does not affect	Does not affect	Does not affect
G11V	Does not affect	Increases	Does not affect	Does not affect
L12I	Decreases	Does not affect	Does not affect	Does not affect
L12V	Decreases	Does not affect	Does not affect	Does not affect
L13Q	Decreases	Does not affect	Does not affect	Does not affect
A14E	Does not affect	Does not affect	Does not affect	Does not affect
P16H	Decreases	Does not affect	Does not affect	Does not affect
P16S	Decreases	Does not affect	Does not affect	Does not affect
S17T	Does not affect	Does not affect	Does not affect	Does not affect
P18A	Decreases	Does not affect	Does not affect	Does not affect
P18T	Decreases	Does not affect	Does not affect	Does not affect
A19S	Does not affect	Does not affect	Does not affect	Does not affect
A21D	Does not affect	Does not affect	Does not affect	Does not affect
A21V	Does not affect	Does not affect	Does not affect	Does not affect
Q22R	Does not affect	Does not affect	Does not affect	Does not affect
T23A	Does not affect	Does not affect	Does not affect	Does not affect
T23N	Does not affect	Does not affect	Does not affect	Does not affect
T23I	Does not affect	Does not affect	Does not affect	Does not affect
T23S	Does not affect	Does not affect	Does not affect	Does not affect
L24F	Decreases	Does not affect	Does not affect	Does not affect
T25A	Does not affect	Does not affect	Does not affect	Does not affect
T25N	Does not affect	Does not affect	Does not affect	Does not affect
R26Q	Does not affect	Does not affect	Does not affect	Does not affect
R26W	Does not affect	Does not affect	Does not affect	Does not affect
V27F	Decreases	Does not affect	Does not affect	Does not affect
P28R	Decreases	Does not affect	Does not affect	Does not affect
R29Q	Does not affect	Does not affect	Does not affect	Does not affect
R29L	Does not affect	Does not affect	Does not affect	Does not affect
P30Q	Decreases	Does not affect	Does not affect	Does not affect
P30L	Does not affect	Does not affect	Does not affect	Does not affect
P30T	Decreases	Does not affect	Does not affect	Does not affect
E32Q	Does not affect	Does not affect	Does not affect	Does not affect
E32K	Decreases	Does not affect	Does not affect	Does not affect
L33V	Decreases	Does not affect	Does not affect	Does not affect
P35L	Does not affect	Does not affect	Does not affect	Does not affect
C37R	Does not affect	Does not affect	Does not affect	Does not affect

G38C	Decreases	Does not affect	Does not affect	Does not affect
G38S	Decreases	Does not affect	Does not affect	Does not affect
R39C	Decreases	Does not affect	Does not affect	Does not affect
R39H	Decreases	Does not affect	Does not affect	Does not affect
R39L	Does not affect	Does not affect	Does not affect	Does not affect
R40C	Decreases	Does not affect	Does not affect	Does not affect
G41R	Does not affect	Does not affect	Does not affect	Does not affect
G41V	Does not affect	Does not affect	Does not affect	Does not affect
L42R	Decreases	Does not affect	Does not affect	Does not affect
R43C	Decreases	Does not affect	Does not affect	Does not affect
R43H	Decreases	Does not affect	Does not affect	Does not affect
R43L	Does not affect	Does not affect	Does not affect	Does not affect
R43P	Does not affect	Does not affect	Does not affect	Does not affect
T44N	Decreases	Does not affect	Does not affect	Does not affect
T44I	Does not affect	Does not affect	Does not affect	Does not affect
T44P	Decreases	Does not affect	Does not affect	Does not affect
D45N	Decreases	Does not affect	Does not affect	Does not affect
D45H	Does not affect	Does not affect	Does not affect	Does not affect
I46N	Decreases	Does not affect	Does not affect	Does not affect
I46M	Decreases	Does not affect	Does not affect	Does not affect
I46S	Decreases	Does not affect	Does not affect	Does not affect
I46T	Decreases	Does not affect	Does not affect	Does not affect
I46V	Decreases	Does not affect	Does not affect	Does not affect
D47E	Does not affect	Does not affect	Does not affect	Does not affect
A48T	Decreases	Does not affect	Does not affect	Does not affect
A48V	Does not affect	Does not affect	Does not affect	Does not affect
T49N	Decreases	Does not affect	Does not affect	Does not affect
T49P	Decreases	Does not affect	Does not affect	Does not affect
T49S	Decreases	Does not affect	Does not affect	Does not affect
C50R	Does not affect	Does not affect	Does not affect	Does not affect
C50F	Does not affect	Does not affect	Does not affect	Does not affect
C50Y	Does not affect	Does not affect	Does not affect	Does not affect
T51A	Decreases	Does not affect	Does not affect	Does not affect
T51M	Does not affect	Does not affect	Does not affect	Does not affect
P52L	Decreases	Does not affect	Does not affect	Does not affect
P52S	Decreases	Does not affect	Does not affect	Does not affect
P52T	Decreases	Does not affect	Does not affect	Does not affect
R53H	Decreases	Does not affect	Does not affect	Does not affect
R53L	Does not affect	Does not affect	Does not affect	Does not affect
R54C	Decreases	Does not affect	Does not affect	Does not affect
R54G	Decreases	Does not affect	Does not affect	Does not affect
R54H	Decreases	Does not affect	Does not affect	Does not affect
A55T	Decreases	Does not affect	Does not affect	Does not affect
S56R	Does not affect	Does not affect	Does not affect	Does not affect
S56N	Does not affect	Does not affect	Does not affect	Does not affect
S57L	Does not affect	Does not affect	Does not affect	Does not affect
Q59H	Decreases	Does not affect	Does not affect	Does not affect
R60C	Decreases	Does not affect	Does not affect	Does not affect
R60H	Decreases	Does not affect	Does not affect	Does not affect
G61S	Decreases	Does not affect	Does not affect	Does not affect
G61V	Decreases	Does not affect	Does not affect	Does not affect
L62P	Decreases	Does not affect	Does not affect	Does not affect
N67S	Does not affect	Does not affect	Does not affect	Does not affect
V68A	Decreases	Does not affect	Does not affect	Does not affect

V68G	Decreases	Does not affect	Does not affect	Does not affect
V68I	Does not affect	Does not affect	Increases	Does not affect
V73A	Decreases	Does not affect	Increases	Does not affect
Y74C	Decreases	Does not affect	Does not affect	Does not affect
M76T	Decreases	Does not affect	Does not affect	Does not affect
M76V	Decreases	Increases	Does not affect	Does not affect
K80N	Does not affect	Does not affect	Does not affect	Does not affect
S81T	Does not affect	Does not affect	Does not affect	Does not affect
L84F	Decreases	Does not affect	Does not affect	Does not affect
L84W	Decreases	Does not affect	Does not affect	Does not affect
G85A	Decreases	Does not affect	Does not affect	Does not affect
G85V	Does not affect	Does not affect	Does not affect	Does not affect
H86P	Does not affect	Does not affect	Does not affect	Does not affect
G88C	Decreases	Does not affect	Does not affect	Does not affect
D91H	Does not affect	Does not affect	Does not affect	Does not affect
E92K	Does not affect	Does not affect	Does not affect	Does not affect
T93I	Does not affect	Does not affect	Does not affect	Does not affect
T94A	Decreases	Does not affect	Does not affect	Does not affect
Y95C	Decreases	Does not affect	Does not affect	Does not affect
Y95H	Decreases	Does not affect	Does not affect	Does not affect
E96K	Does not affect	Does not affect	Does not affect	Does not affect
R97G	Decreases	Does not affect	Does not affect	Does not affect
R97K	Does not affect	Does not affect	Does not affect	Does not affect
A99T	Decreases	Does not affect	Does not affect	Does not affect
E100A	Does not affect	Does not affect	Does not affect	Does not affect
T102M	Does not affect	Does not affect	Does not affect	Does not affect
D104E	Does not affect	Does not affect	Does not affect	Does not affect
S105F	Increases	Does not affect	Does not affect	Does not affect
L106S	Decreases	Does not affect	Does not affect	Does not affect
L106V	Decreases	Does not affect	Does not affect	Does not affect
A107P	Does not affect	Does not affect	Does not affect	Does not affect
E108D	Decreases	Does not affect	Does not affect	Does not affect
E108V	Does not affect	Increases	Increases	Does not affect
F110S	Decreases	Does not affect	Does not affect	Does not affect
D112A	Does not affect	Does not affect	Does not affect	Does not affect
D112H	Does not affect	Does not affect	Does not affect	Does not affect
D112Y	Does not affect	Does not affect	Increases	Does not affect
L113I	Decreases	Does not affect	Does not affect	Does not affect
A114V	Does not affect	Does not affect	Does not affect	Does not affect
D115E	Does not affect	Does not affect	Does not affect	Does not affect
K116E	Does not affect	Does not affect	Does not affect	Does not affect
P117L	Does not affect	Does not affect	Does not affect	Does not affect
P117T	Decreases	Does not affect	Does not affect	Does not affect
Y118C	Decreases	Does not affect	Does not affect	Does not affect
T119K	Decreases	Does not affect	Does not affect	Does not affect
T119M	Does not affect	Does not affect	Does not affect	Does not affect
D122Y	Does not affect	Does not affect	Increases	Does not affect
Y123F	Does not affect	Does not affect	Does not affect	Does not affect
G130A	Decreases	Increases	Does not affect	Does not affect
G130S	Decreases	Does not affect	Does not affect	Does not affect
G130V	Does not affect	Increases	Does not affect	Does not affect
T133A	Decreases	Does not affect	Does not affect	Does not affect
V134G	Decreases	Does not affect	Does not affect	Does not affect
V134I	Decreases	Does not affect	Does not affect	Does not affect

K135R	Does not affect	Does not affect	Does not affect	Does not affect
G138R	Does not affect	Decreases	Does not affect	Does not affect
D139Y	Does not affect	Does not affect	Does not affect	Does not affect
D139V	Does not affect	Increases	Does not affect	Does not affect
N146K	Does not affect	Decreases	Does not affect	Does not affect
Q148R	Does not affect	Does not affect	Does not affect	Does not affect
T149A	Decreases	Does not affect	Does not affect	Does not affect
T149M	Does not affect	Does not affect	Does not affect	Does not affect
K152E	Does not affect	Does not affect	Does not affect	Does not affect
Q153R	Does not affect	Does not affect	Does not affect	Does not affect
Q153H	Decreases	Does not affect	Does not affect	Does not affect
I154F	Decreases	Does not affect	Does not affect	Does not affect
I154V	Decreases	Does not affect	Does not affect	Does not affect
W155R	Decreases	Does not affect	Does not affect	Does not affect
L156I	Decreases	Does not affect	Increases	Does not affect
L156P	Decreases	Does not affect	Does not affect	Does not affect
S158A	Decreases	Does not affect	Does not affect	Does not affect
S158P	Does not affect	Does not affect	Does not affect	Does not affect
S160C	Decreases	Does not affect	Does not affect	Does not affect
S161R	Does not affect	Does not affect	Does not affect	Does not affect
S161T	Does not affect	Does not affect	Does not affect	Does not affect
K164R	Does not affect	Does not affect	Does not affect	Does not affect
R165C	Decreases	Does not affect	Does not affect	Does not affect
R165H	Decreases	Does not affect	Does not affect	Does not affect
R165P	Decreases	Does not affect	Does not affect	Does not affect
Y166F	Decreases	Does not affect	Does not affect	Does not affect
G170W	Does not affect	Does not affect	Does not affect	Does not affect
K171R	Does not affect	Does not affect	Does not affect	Does not affect
K171E	Does not affect	Does not affect	Does not affect	Does not affect
W173G	Decreases	Does not affect	Does not affect	Does not affect
V174A	Decreases	Does not affect	Increases	Does not affect
V174L	Decreases	Does not affect	Increases	Does not affect
Y175F	Does not affect	Does not affect	Does not affect	Does not affect
H177Y	Does not affect	Increases	Does not affect	Does not affect
D178N	Decreases	Does not affect	Does not affect	Does not affect
D178E	Does not affect	Does not affect	Does not affect	Does not affect
G179S	Decreases	Does not affect	Does not affect	Does not affect
V180M	Decreases	Does not affect	Does not affect	Does not affect
S181C	Does not affect	Does not affect	Does not affect	Does not affect
L182H	Decreases	Does not affect	Does not affect	Does not affect
L182F	Decreases	Does not affect	Does not affect	Does not affect
H183R	Does not affect	Does not affect	Does not affect	Does not affect
H183L	Increases	Does not affect	Does not affect	Does not affect
H183Y	Does not affect	Does not affect	Increases	Does not affect
L186R	Decreases	Does not affect	Does not affect	Does not affect
A187V	Does not affect	Does not affect	Does not affect	Does not affect
A188T	Does not affect	Does not affect	Does not affect	Does not affect
T191A	Does not affect	Does not affect	Does not affect	Does not affect
T191S	Does not affect	Does not affect	Does not affect	Does not affect
A193P	Does not affect	Does not affect	Does not affect	Does not affect
T196A	Decreases	Does not affect	Does not affect	Does not affect
K197R	Does not affect	Does not affect	Does not affect	Does not affect
L198R	Decreases	Does not affect	Does not affect	Does not affect
L198V	Decreases	Does not affect	Does not affect	Does not affect

D199N	Decreases	Does not affect	Does not affect	Does not affect
D199G	Decreases	Does not affect	Does not affect	Does not affect
L200S	Decreases	Does not affect	Does not affect	Does not affect
S202C	Does not affect	Does not affect	Does not affect	Does not affect
S202P	Does not affect	Does not affect	Does not affect	Does not affect
L203F	Decreases	Does not affect	Does not affect	Does not affect
A204P	Does not affect	Does not affect	Does not affect	Does not affect
S206C	Does not affect	Does not affect	Does not affect	Does not affect
S206T	Does not affect	Does not affect	Does not affect	Does not affect
S206Y	Does not affect	Does not affect	Does not affect	Does not affect
G207R	Does not affect	Does not affect	Does not affect	Does not affect
D209G	Decreases	Does not affect	Does not affect	Does not affect
