# Supplementary material for: An In Silico Analysis of Genetic Variants and Structural Modeling of the Human Frataxin Protein in Friedreich’s Ataxia
Source: Int J Mol Sci. 2024 May 26;25(11):5796. doi: 10.3390/ijms25115796 (PMC11172458; doi:10.3390/ijms25115796)
Supplement: Supplementary file 1 [file ijms-25-05796-s001.zip › Table S4.pdf]

Table S4. Evolutionary conservation analysis of FXN protein variants.

Variants	Position	ConSurf-score
M1V	1	8
M1I	1	8
M1L	1	8
M1T	1	8
M1K	1	8
W2L	2	6
L4P	4	3
G5R	5	6
R6H	6	4
R6L	6	4
R7H	7	7
A8T	8	5
A8G	8	5
V9I	9	5
A10T	10	4
A10V	10	4
G11S	11	2
G11V	11	2
L12V	12	4
L12I	12	4
L13Q	13	7
A14E	14	5
P16S	16	4
P16H	16	4
S17T	17	2
P18A	18	1
P18T	18	1
A19S	19	2
A21V	21	4
A21D	21	4
Q22R	22	1
T23A	23	6
T23I	23	6
T23N	23	6
T23S	23	6
L24F	24	1
T25A	25	3
T25N	25	3
R26W	26	1
R26Q	26	1
V27F	27	3
P28R	28	1
R29Q	29	1
R29L	29	1
P30L	30	1
P30Q	30	1

P30T	30	1
E32K	32	2
E32Q	32	2
L33V	33	1
P35L	35	1
C37R	37	1
G38C	38	1
G38S	38	1
R39C	39	1
R39H	39	1
R39L	39	1
R40C	40	3
G41R	41	1
G41V	41	1
L42R	42	3
R43L	43	1
R43C	43	1
R43P	43	1
R43H	43	1
T44P	44	2
T44N	44	2
T44I	44	2
D45N	45	1
D45H	45	1
I46V	46	4
I46T	46	4
I46N	46	4
I46S	46	4
I46M	46	4
D47E	47	1
A48T	48	1
A48V	48	1
T49S	49	3
T49P	49	3
T49N	49	3
C50F	50	1
C50Y	50	1
C50R	50	1
T51M	51	1
T51A	51	1
P52T	52	1
P52L	52	1
P52S	52	1
R53H	53	1
R53L	53	1
R54C	54	1
R54G	54	1
R54H	54	1
A55T	55	1

S56N	56	5
S56R	56	5
S57L	57	2
Q59H	59	1
R60C	60	1
R60H	60	1
G61S	61	1
G61V	61	1
L62P	62	6
N67S	67	1
V68I	68	2
V68G	68	2
V68A	68	2
V73A	73	6
Y74C	74	1
M76T	76	2
M76V	76	2
K80N	80	3
S81T	81	1
L84F	84	1
L84W	84	1
G85V	85	2
G85A	85	2
H86P	86	3
G88C	88	6
D91H	91	9
E92K	92	7
T93I	93	7
T94A	94	5
Y95H	95	8
Y95C	95	8
E96K	96	9
R97K	97	7
R97G	97	7
A99T	99	9
E100A	100	8
T102M	102	9
D104E	104	8
S105F	105	7
L106S	106	9
L106V	106	9
A107P	107	8
E108V	108	7
E108D	108	7
F110S	110	8
D112Y	112	9
D112H	112	9
D112A	112	9
L113I	113	9

A114V	114	6
D115E	115	8
K116E	116	7
P117T	117	7
P117L	117	7
Y118C	118	6
T119M	119	8
T119K	119	8
D122Y	122	8
Y123F	123	7
G130V	130	9
G130A	130	9
G130S	130	9
T133A	133	9
V134G	134	5
V134I	134	5
K135R	135	8
G138R	138	5
D139Y	139	5
D139V	139	5
N146K	146	9
Q148R	148	9
T149A	149	9
T149M	149	9
K152E	152	6
Q153R	153	8
Q153H	153	8
I154F	154	9
I154V	154	9
W155R	155	8
L156P	156	9
L156I	156	9
S158P	158	9
S158A	158	9
S160C	160	6
S161T	161	8
S161R	161	8
K164R	164	9
R165P	165	7
R165H	165	7
R165C	165	7
Y166F	166	8
G170W	170	9
K171R	171	4
K171E	171	4
W173G	173	7
V174A	174	8
V174L	174	8
Y175F	175	9

H177Y	177	9
D178E	178	8
D178N	178	8
G179S	179	7
V180M	180	2
S181C	181	8
L182F	182	9
L182H	182	9
H183L	183	9
H183R	183	9
H183Y	183	9
L186R	186	9
A187V	187	6
A188T	188	2
T191S	191	9
T191A	191	9
A193P	193	8
T196A	196	6
K197R	197	3
L198V	198	5
L198R	198	5
D199G	199	9
D199N	199	9
L200S	200	7
S202P	202	3
S202C	202	3
L203F	203	9
A204P	204	4
S206T	206	9
S206C	206	9
S206Y	206	9
G207R	207	7
D209G	209	1
