# Supplementary figures and images for: An In Silico Analysis of Genetic Variants and Structural Modeling of the Human Frataxin Protein in Friedreich’s Ataxia
Source: Int J Mol Sci. 2024 May 26;25(11):5796. doi: 10.3390/ijms25115796 (PMC11172458; doi:10.3390/ijms25115796)

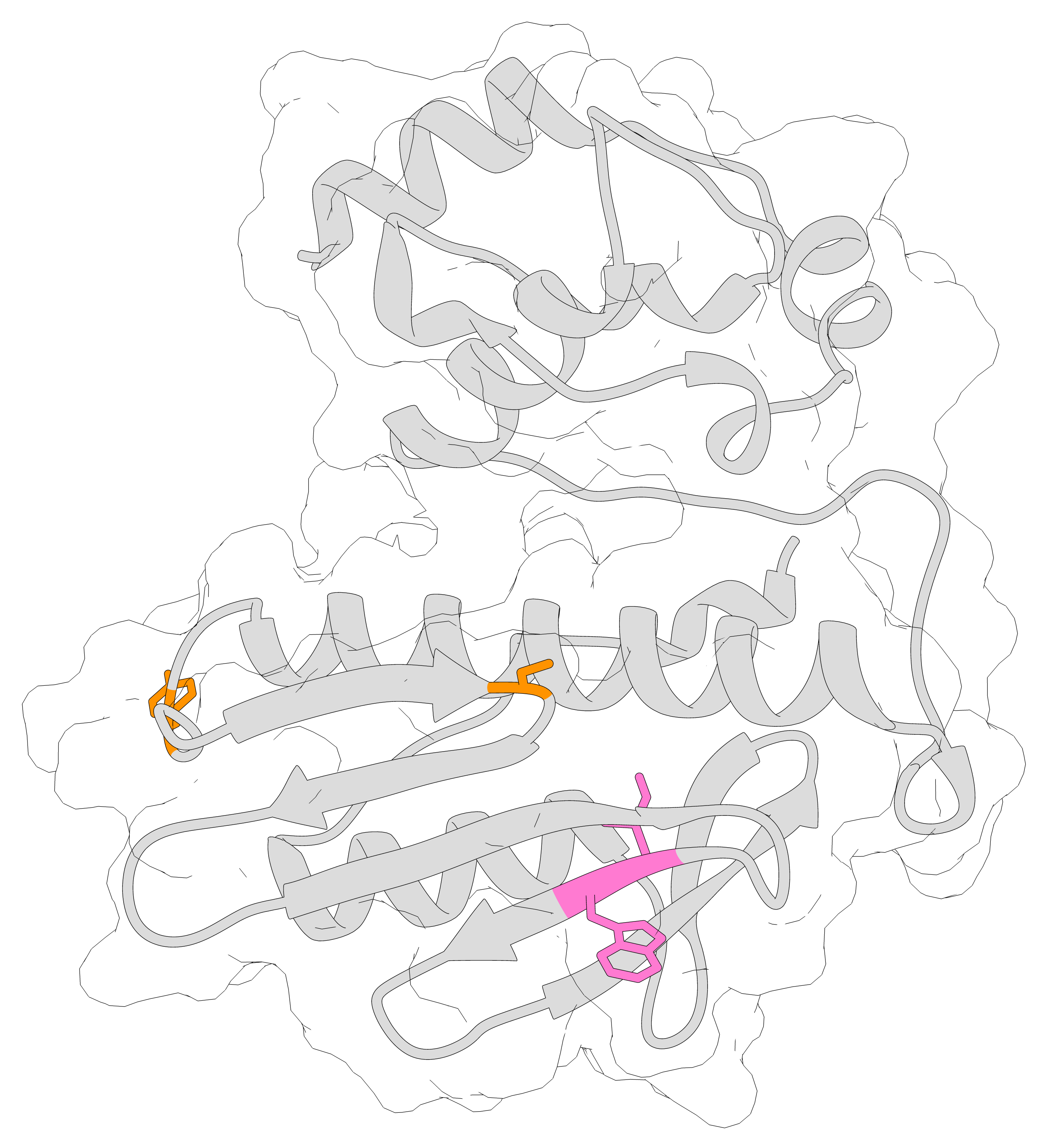

Supplement: Supplementary file 1 [file ijms-25-05796-s001.zip › Figure S1.png]

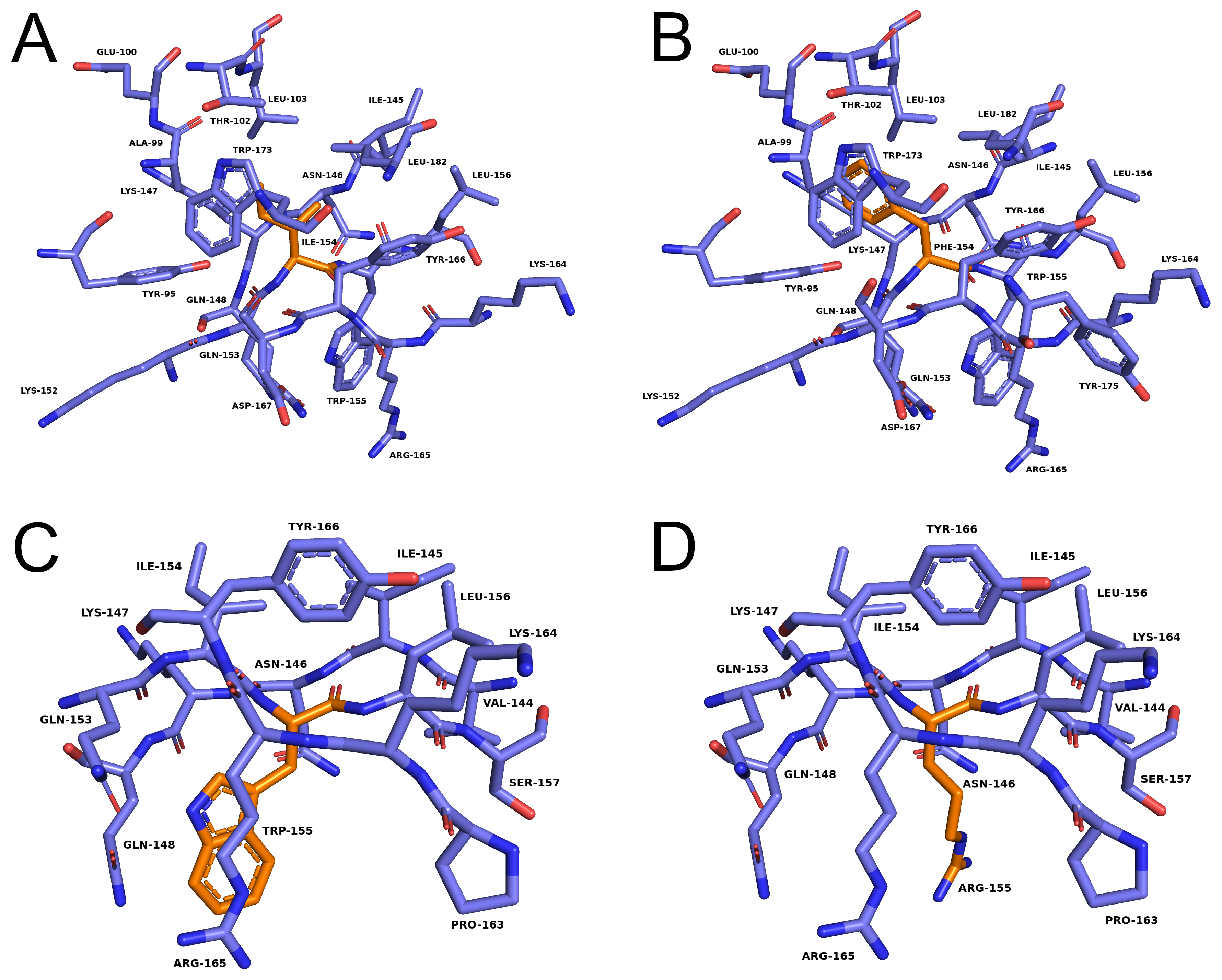

Supplement: Supplementary file 1 [file ijms-25-05796-s001.zip › Figure S2.png]

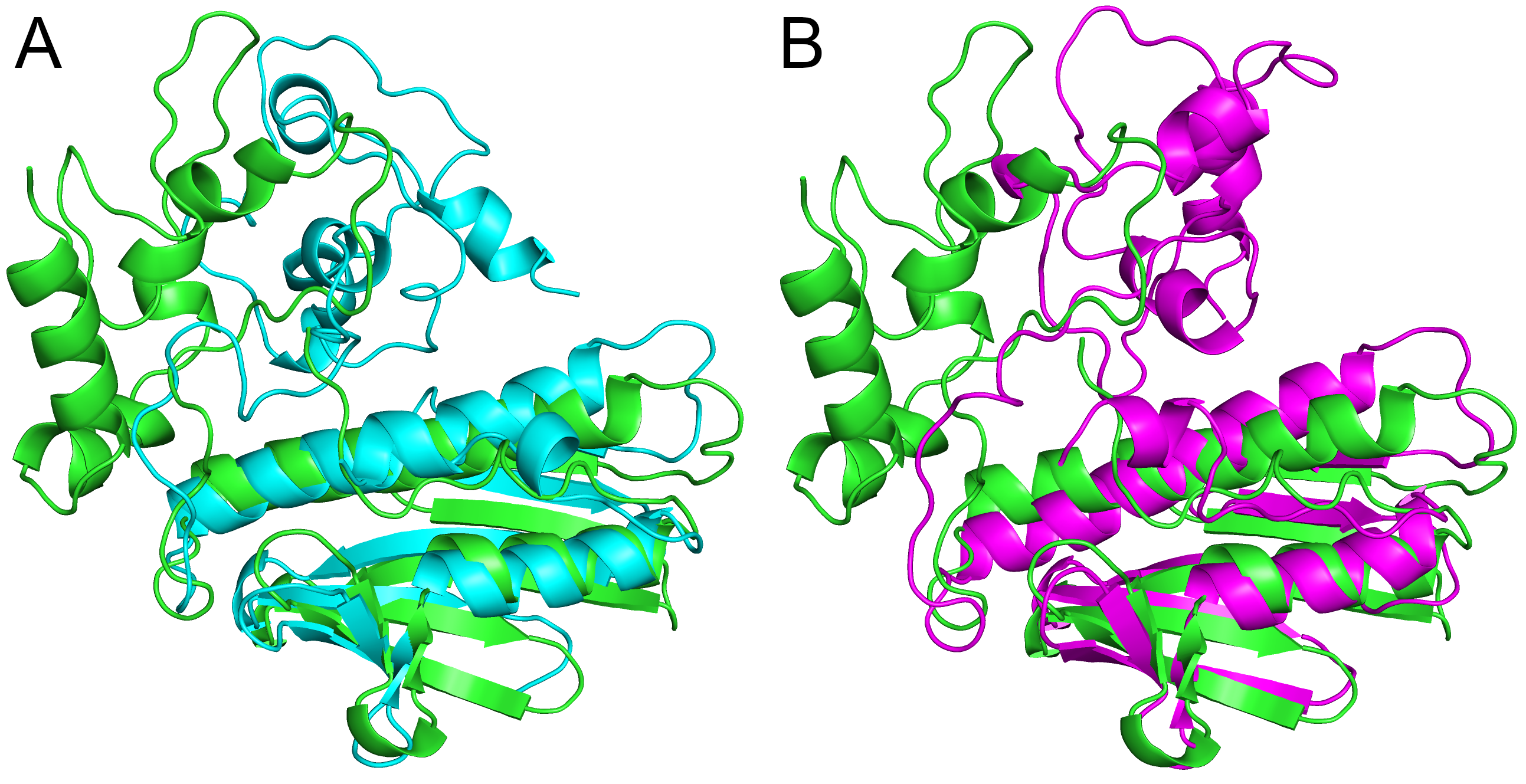

Supplement: Supplementary file 1 [file ijms-25-05796-s001.zip › Figure S3.png]
